# A comprehensive meta QTL analysis for fiber quality, yield, yield related and morphological traits, drought tolerance, and disease resistance in tetraploid cotton

**DOI:** 10.1186/1471-2164-14-776

**Published:** 2013-11-11

**Authors:** Joseph I Said, Zhongxu Lin, Xianlong Zhang, Mingzhou Song, Jinfa Zhang

**Affiliations:** 1 Department of Plant and Environmental Sciences, New Mexico State University, Las Cruces, NM, USA; 2 National Key Laboratory of Crop Genetic Improvement & National Centre of Plant Gene Research (Wuhan), Huazhong Agricultural University, Wuhan 430070, Hubei, People’s Republic of China; 3 Department of Computer Science, New Mexico State University, Las Cruces, NM, USA

## Abstract

**Background:**

The study of quantitative trait loci (QTL) in cotton (*Gossypium* spp.) is focused on traits of agricultural significance. Previous studies have identified a plethora of QTL attributed to fiber quality, disease and pest resistance, branch number, seed quality and yield and yield related traits, drought tolerance, and morphological traits. However, results among these studies differed due to the use of different genetic populations, markers and marker densities, and testing environments. Since two previous meta-QTL analyses were performed on fiber traits, a number of papers on QTL mapping of fiber quality, yield traits, morphological traits, and disease resistance have been published. To obtain a better insight into the genome-wide distribution of QTL and to identify consistent QTL for marker assisted breeding in cotton, an updated comparative QTL analysis is needed.

**Results:**

In this study, a total of 1,223 QTL from 42 different QTL studies in *Gossypium* were surveyed and mapped using Biomercator V3 based on the *Gossypium* consensus map from the Cotton Marker Database. A meta-analysis was first performed using manual inference and confirmed by Biomercator V3 to identify possible QTL clusters and hotspots. QTL clusters are composed of QTL of various traits which are concentrated in a specific region on a chromosome, whereas hotspots are composed of only one trait type. QTL were not evenly distributed along the cotton genome and were concentrated in specific regions on each chromosome. QTL hotspots for fiber quality traits were found in the same regions as the clusters, indicating that clusters may also form hotspots.

**Conclusions:**

Putative QTL clusters were identified via meta-analysis and will be useful for breeding programs and future studies involving *Gossypium* QTL. The presence of QTL clusters and hotspots indicates consensus regions across cultivated tetraploid *Gossypium* species, environments, and populations which contain large numbers of QTL, and in some cases multiple QTL associated with the same trait termed a hotspot. This study combines two previous meta-analysis studies and adds all other currently available QTL studies, making it the most comprehensive meta-analysis study in cotton to date.

## Background

The *Gossypium* genus is composed of approximately 50 species including four cultivated ones which vary in morphological and economic characteristics considerably [1]. Species which possess superior fiber and yield traits are tetraploids, *Gossypium hirsutum* and *G*. *barbadense*. *G. hirsutum,* also called Upland cotton, is grown in warm climates worldwide, and possesses high lint yield which is vital to the textile industry. *G. barbadense,* also called Egyptian cotton, Pima cotton, or Sea-island cotton, is known for superior fiber length, strength, and fineness [1]. Both species have been the focus of breeding programs to combine their superior fiber quantity and quality traits [1] since the rediscovery of Mendelian genetics more than a century ago.

In cotton and other crop species quantitative traits are governed by a multitude of loci each of which contributes to a particular phenotype in varying degrees [2]. These loci are called quantitative trait loci (QTL) and are evaluated for their positions on the chromosomes, and the phenotypic variance that they contribute to a particular trait [2]. Studies in *Gossypium* have focused on QTL which are involved in fiber strength, length, uniformity, micronaire, color, disease resistance, fruiting nodes, boll weight and number, yield, seed oil and protein content, leaf morphology, and various seed related traits [3,4]. Numerous independent studies have reported QTL pertaining to all of these traits using independent or updated linkage maps from different or the same segregating populations.

Historically QTL mapping studies were expensive undertakings that may have yielded little to no results. Creation of sufficient markers to saturate the genome was expensive with no guarantee that the markers generated would be anywhere close to the QTL of interest [5]. Marker development has improved significantly and has become much less expensive [6]. Mapping strategies have also progressed from simple interval mapping (SIM) to more complex composite interval mapping (CIM), multiple QTL mapping (MQM), and others which greatly increase the accuracy of placing QTL [7]. This study uses a mixture of more advanced mapping techniques including CIM and MQM.

QTL mapping is an important tool for breeders to combine economically important traits together to create a superior cultivar. Crosses between *G. barbadense* and *G. hirsutum* have been attempted for decades to combine their superior fiber quality and yield traits. While the two species are genetically close enough to be crossed there are issues with sterility, cytological abnormalities, and distorted segregation [4,8]. Differences in mapping populations, genotypes, and environments can yield heterogeneous results in QTL mapping [3,8]. For this reason, meta-analysis is useful in marker assisted selection as it merges datasets and creates consensus map positions for QTL. Not only does meta-analysis help to confirm the existence of declared QTL from various studies by creating possible hotspots where QTL from the same trait aggregate but it can imply the existence of pleotropic traits by creating QTL clusters for various traits.

Previous QTL mapping studies, 42 of which were cumulatively used in this study are summarized in Table 1[4,9-49]. Fiber quality traits which are of upmost importance to cotton breeding programs have dominated these studies in *Gossypium* compared to yield and disease resistance related traits [3,4]. For this reason, the majority of the QTL in this study are fiber quality related QTL. Studies done thus far have varied in linkage groups used, mapping techniques, *Gossypium* species, mapping populations, and markers. It is important to consolidate all of the studies into a consensus map to identify possible QTL clusters and hotspots.

**Table 1:** The author, journal, publication year, number of QTL used in this study, and population data

**Author**	**Journal**	**Year**	**# of QTL**	**Population**	**Trait type**
An C et al	Euphytica	2010	26	F2	Yield
Chee P et al	Theor Appl Genet	2005	17	BC3F2	Fiber
Chen H et al	Theor Appl Genet	2009	20	RIL	Fiber
Draye X et al	Theor Appl Genet	2005	36	BC3F2	Fiber
Feng J et al	Sci China Series C: Life Sciences	2009	41	F2:3	Resistance
Guo Y et al	Euphytica	2008	5	F2	Morphological
Gutierrez OA et al	Theor Appl Genet	2010	12	RIL	Resistance
Gutierrez OA et al	Theor Appl Genet	2011	3	BCP1/2	Resistance
Jiang C et al	Theor Appl Genet	2000	16	F2	Morpholigical
Jiang CX et al	Proc. Natl. Acad. Sci. USA	1998	7	F2	Fiber
Lacape JM et al	BMC Plant Biology	2010	233	RIL	Fiber
Lacape JM et al	Crop Science	2005	61	BC1,2,BC2S1	Fiber
Li C et al	Euphytica	2012	7	F2:3	Morpholigical
Liu HY et al	Euphytica	2012	10	RIL	Yield
Liu R et al	Mol Breed	2012	26	RIL	Yield
Mei M et al	Theor Appl Genet	2004	3	F2	Fiber
Paterson AH et al	Theor Appl Genet	2003	24	F2/F3	Fiber
Qin H et al	Theor Appl Genet	2008	20	4WC	Yield and Fiber
Rong J et al	Theor Appl Genet	2005	5	F2	Yield
Saranga Y et al	Plant Cell Environ	2004	35	F2/F3	Yield
Shen X et al	Mol Breed	2005	28	F2 & F2:3	Fiber
Shen X et al	Crop Sci	2006	1	RIL	Yield
Shen X et al	Theor Appl Genet	2006	13	F2	Resistance
Shen X et al	Theor Appl Genet	2010	1	F2	Resistance
Sun FD et al	Mol Breed	2012	39	RIL	Fiber
Waghmare VN et al	Theor Appl Genet	2005	9	F2	Morpholigical
Wang B et al	Euphytica	2006	24	RIL	Fiber
Wang C et al	PLoS ONE	2012	45	RIL	Resistance
Wang F et al	Mol Breed	2013	21	F2/F2:3	Fiber
Wang HM et al	Journal Integr Plant Biol	2008	4	F2:3	Resistance
Wang P et al	Theor Appl Genet	2012	33	CSIL	Fiber
Wang P et al	Theor Appl Genet	2009	2	F2:3	Resistance
Wright RJ et al	J Hered	1999	6	B2/B3b6/B3	Morpholigical
Wright RJ et al	Genetics	1998	2	B2/B3b6/B3	Resistance
Wu Jixiang et al	Euphytica	2009	56	RIL	Fiber
Yang C et al	Plant Sci	2008	18	BC1S2	Resistance
Yu J et al	Euphytica	2012	41	BIL	Seed
Yu J et al	Euphytica	2013	103	F2:F2:3:TC	Fiber
Yu J et al	Theor Appl Genet	2013	67	BIL	Fiber and Yield
Zhang K et al	Mol Breed	2012	60	F1:2,1:3	Fiber
Zhang Z et al	Theor Appl Genet	2011	30	BC3F1	Fiber
Zhang ZS et al	Mol Breed	2009	13	RIL	Fiber

Two large scale meta-analyses of *Gossypium* were conducted by Rong et al. [3] and Lacape et al. [4]. The study by Lacape et al. focused on fiber quality traits, while Rong et al. [3] study was more comprehensive where the traits included fiber and seed, quality, drought tolerance to leaf morphology, and bacterial blight (Xcm) resistance. Lacape et al.’s [4] study on fiber quality was based on a *G. barbadense* x *G. hirsutum* (Gb x Gh hereafter) recombinant inbred line (RIL) population, whereas Rong et al.’s study pooled QTL from a multitude of segregating populations (F2, F2:3, or BC2) tested in a single environment with no replicates. The study by Rong et al. [3] pooled a variety of QTL trait types but lacked sufficient QTL from any one trait to declare hotspots. Since Rong et al.’s [3] work in 2007, many new yields and resistance related QTL have been described by numerous publications, not included in Lacape et al’s [4] work. This study combines the work from the two meta-QTL studies and numerous other recent publications to perform a meta-analysis based on 42 different studies using a total of 1,223 QTL. Furthermore, some declared clusters in Rong et al.’s [3] study consisted of only two QTL, whereas clusters in this study required four or more QTL to declare a cluster. Placing more stringent requirements on what constitutes a cluster decreases the chances of declaring a false positive cluster. Also with the addition of newly discovered QTL declaring clusters with so few QTL would place a cluster on nearly every segment of every chromosome. In this study, the majority of clusters contains far more than four QTL and signifies a heavily populated region of QTL on the chromosome. With the addition of new QTL previously not described by meta-analysis, many hotspot clusters were identified in this study which were previously unknown. This study provides a comprehensive and updated meta-analysis of *Gossypium*.

## Results

### QTL Distribution

All published QTL in the study along with their population types, trait types, authors, journals, and publication years are described in Table 1[4,9-49]. The distribution of QTL over the genome is not uniform, some chromosomes contain more QTL than others, and different combinations of QTL. The distributions of QTL on each chromosome for each trait type are described in Table 2. QTL clusters and hotspots are described in Table 3, and hotspots are shown in Additional file 1: Figures S3–S11 which pertain to fiber quality, *Verticillium* resistance, nematode resistance, and leaf morphology QTL hotspots. The majority of hotspots was fiber quality QTL, and of those most pertained to micronaire QTL. QTL clusters appeared on every chromosome; however, some chromosomes were more densely populated with QTL and contained more clusters than others. Table 2 offers a comprehensive overview of all QTL in the study and their distribution over the genome. The following is a summary and description of all traits and significant chromosomes containing the most QTL of that particular trait.

**Table 2:** Distribution of fiber quality, yield, seed, leaf morphology and resistance QTL across the cotton genome

**Fiber quality QTL traits**									
**Chromosome**	**FS**	**FL**	**Micro**	**FU**	**FE**	**Color**	**FM**	**Perimeter**	
c1	5	8	11	1	5	3	0	0	
c2	3	0	6	5	6	0	0	0	
c3	5	16	11	1	2	1	1	0	
c4	2	7	7	3	1	0	0	0	
c5	6	4	16	4	2	2	1	0	
c6	1	6	12	3	3	8	0	0	
c7	9	7	4	3	5	2	0	0	
c8	2	5	3	3	1	9	0	0	
c9	2	5	9	3	5	6	1	0	
c10	1	4	8	2	4	1	1	1	
c11	5	1	2	0	0	3	0	0	
c12	7	9	11	10	7	0	1	0	
c13	1	3	4	1	3	0	0	0	
c14	9	8	13	5	8	4	2	1	
c15	3	4	13	5	9	2	0	0	
c16	10	2	10	4	2	1	1	0	
c17	0	1	7	7	4	2	0	0	
c18	6	6	9	4	4	4	1	0	
c19	2	9	10	2	12	3	0	0	
c20	2	4	5	4	6	0	0	0	
c21	5	6	9	3	4	2	0	0	
c22	1	0	3	4	2	2	0	0	
c23	13	10	6	1	8	4	0	0	
c24	26	5	17	6	9	0	1	0	
c25	4	7	20	2	3	11	1	0	
c26	2	14	8	5	3	1	0	0	
**Total**	**132**	**151**	**234**	**91**	**118**	**71**	**11**	**2**	
**Fiber quality and yield QTL traits**									
**Chromosome**	**WT**	**Wall thick**	**SLF**	**HI**	**BW**	**LI**	**LP**	**SCY**	
c1	0	0	0	0	1	0	0	1	
c2	0	0	0	2	1	0	0	2	
c3	0	1	0	0	0	0	1	1	
c4	0	0	0	0	1	1	1	0	
c5	0	1	0	0	2	1	2	0	
c6	0	0	0	0	0	0	0	1	
c7	0	0	0	0	0	1	3	1	
c8	0	0	0	0	0	0	0	0	
c9	0	1	0	0	0	1	1	3	
c10	1	0	0	0	0	1	1	1	
c11	0	0	0	0	1	2	3	1	
c12	0	0	0	0	1	2	2	2	
c13	0	0	0	0	0	0	0	1	
c14	1	0	0	1	4	2	1	3	
c15	0	0	0	0	1	0	1	1	
c16	0	0	0	0	1	0	2	1	
c17	0	0	0	0	0	0	1	0	
c18	0	1	1	2	3	0	0	3	
c19	0	0	0	0	0	0	0	0	
c20	1	0	0	0	0	0	0	1	
c21	0	0	0	0	1	0	0	0	
c22	0	0	1	0	3	1	0	0	
c23	0	0	0	0	1	0	3	0	
c24	0	0	0	0	1	0	0	1	
c25	0	0	0	0	2	1	0	2	
c26	0	0	0	0	2	2	3	3	
**Total**	**3**	**4**	**2**	**5**	**26**	**15**	**25**	**29**	
**Yield and seed QTL traits**									
**Chromosome**	**LY**	**BN**	**LB**	**Gossypol**	**Protein**	**Oil**	**HP**	**EPP**	**LargenumFS**
c1	3	0	0	0	0	1	0	0	0
c2	0	1	0	0	2	0	0	0	0
c3	1	0	0	1	3	1	1	0	0
c4	0	0	0	0	1	0	0	0	0
c5	1	0	0	0	3	1	0	0	0
c6	1	0	0	0	2	0	0	1	0
c7	2	0	0	0	0	0	0	0	0
c8	0	0	0	0	0	0	0	0	0
c9	1	0	0	0	0	0	0	0	0
c10	0	0	0	0	0	0	0	0	0
c11	0	1	0	0	0	1	0	0	0
c12	1	0	0	0	2	2	0	0	1
c13	2	0	0	1	0	0	0	0	0
c14	3	1	0	0	1	0	1	0	0
c15	1	0	0	0	2	1	0	1	0
c16	1	0	0	0	1	1	0	0	0
c17	0	0	0	0	0	0	0	0	0
c18	2	0	0	1	0	0	0	0	0
c19	0	0	0	2	3	3	0	0	0
c20	1	0	0	0	2	1	0	0	0
c21	0	1	0	0	2	3	0	0	0
c22	1	0	0	1	1	0	0	0	0
c23	0	0	1	0	0	0	0	0	0
c24	1	0	0	0	2	1	0	0	0
c25	0	0	1	0	2	0	0	0	0
c26	1	0	0	0	0	0	0	0	0
**Total**	**23**	**4**	**2**	**6**	**29**	**16**	**2**	**2**	**1**
**Seed quality QTL traits**									
**Chromosome**	**NOFuzFib**	**SW**	**SI**	**SM**					
c1	0	0	0	0					
c2	0	0	0	1					
c3	0	0	1	0					
c4	0	0	0	0					
c5	0	0	0	0					
c6	0	0	0	0					
c7	0	1	1	0					
c8	0	0	0	0					
c9	0	2	0	0					
c10	0	0	0	0					
c11	0	1	0	0					
c12	2	0	0	0					
c13	0	0	0	0					
c14	0	0	3	0					
c15	0	0	0	0					
c16	0	1	0	0					
c17	0	0	1	0					
c18	0	0	0	0					
c19	0	0	0	0					
c20	0	0	0	0					
c21	0	0	0	0					
c22	0	0	1	0					
c23	0	0	1	0					
c24	0	0	1	0					
c25	0	0	0	0					
c26	0	0	1	0					
**Total**	**2**	**5**	**10**	**1**					
**Morphological QTL traits**									
**Chromosome**	**FB num**	**FB node**	**Pubescence**	**NFFB**	**HNFFB**	**Leaf morph**			
c1	0	0	1	1	0	2			
c2	0	0	0	0	0	1			
c3	0	0	0	0	0	1			
c4	0	0	0	0	0	1			
c5	1	0	0	1	0	0			
c6	0	0	2	1	0	3			
c7	0	0	0	0	0	0			
c8	0	0	0	0	0	0			
c9	0	0	0	0	0	2			
c10	0	0	0	0	0	1			
c11	1	0	0	1	0	0			
c12	0	0	0	0	0	1			
c13	0	0	0	0	0	0			
c14	1	0	0	0	0	0			
c15	0	1	0	0	0	6			
c16	1	2	0	0	0	0			
c17	0	0	0	2	1	4			
c18	0	0	0	0	0	1			
c19	0	0	0	0	0	0			
c20	0	0	0	0	0	0			
c21	0	1	0	0	0	0			
c22	0	0	0	0	0	1			
c23	0	0	1	0	0	0			
c24	0	0	0	0	0	0			
c25	0	1	2	0	0	1			
c26	0	0	0	0	0	0			
**Total**	**4**	**5**	**6**	**6**	**1**	**25**			
**Abiotic and biotic resistance QTL traits**									
**Chromosome**	**OP**	**Nematode related**	**VW**	**Fusarium**	**Xcm**				
c1	2	1	0	0	0				
c2	1	0	0	1	0				
c3	0	2	0	0	0				
c4	0	2	0	0	0				
c5	0	7	6	0	1				
c6	1	0	0	0	0				
c7	0	13	2	0	0				
c8	0	0	2	0	0				
c9	0	4	1	0	0				
c10	0	0	0	0	0				
c11	0	17	0	0	0				
c12	0	0	0	0	0				
c13	0	0	0	0	0				
c14	0	8	0	0	1				
c15	0	4	0	1	0				
c16	0	0	16	0	0				
c17	0	0	0	0	0				
c18	0	1	0	0	0				
c19	0	6	3	0	0				
c20	0	3	0	0	0				
c21	0	3	2	0	0				
c22	0	1	2	0	0				
c23	0	2	26	0	0				
c24	0	0	1	0	0				
c25	1	0	0	0	0				
c26	0	0	2	0	0				
**Total**	**5**	**74**	**63**	**2**	**2**				
**Physiological and drought tolerance QTL traits**									
**Chromosome**	**Chlorophyll**	**CIR**	**CT**						
c1	0	0	0						
c2	2	0	0						
c3	0	0	0						
c4	0	0	0						
c5	0	0	0						
c6	0	0	1						
c7	0	0	0						
c8	0	0	0						
c9	0	0	0						
c10	0	0	0						
c11	0	0	0						
c12	0	0	0						
c13	0	0	0						
c14	1	1	0						
c15	0	1	0						
c16	0	0	0						
c17	0	1	0						
c18	0	0	0						
c19	0	0	0						
c20	0	0	0						
c21	0	0	0						
c22	0	0	0						
c23	0	0	0						
c24	0	0	0						
c25	0	1	0						
c26	0	0	0						
**Total**	**3**	**4**	**1**						

The total QTL distributions on the genome are summarized below. The fiber quality, yield, seed, leaf morphology, and resistance related traits are summarized below.

**Table 3:** Distribution of clusters and hotspots over genome

**Chromosome**	**Cluster name**	**Approximate position on chromosome (cM)**	**# of QTL**	**Hotspot name**	**Approximate position on chromosome (cM)**	**# of QTL**
c1	c1-cluster-1	14-34	9	c1-FL-Hotspot-1	66-86	5
	c1-cluster-2	58-82	10			
	c1-cluster-3	95-126	7			
c2	c2-cluster-1	0-14	5	c2-FE-Hotspot-1	30-43	4
	c2-cluster-2	32-52	13			
	c2-cluster-3	82-105	5			
c3	c3-cluster-1	0-20	15	c3-FL-Hotspot-2	0-20	7
	c3-cluster-2	23-48	23	c3-FL-Hotspot-3	43-55	5
				c3-Micronaire-Hotspot-1	23-43	5
c4	c4-cluster-1	0-8	7	c4-FL-Hotspot-4	46-58	5
	c4-cluster-2	46-66	10	c4-Micronaire-Hotspot-2	34-55	4
c5	c5-cluster-1	0-20	15	c5-Micronaire-Hotspot-3	0-20	5
	c5-cluster-2	22-42	16	c5-Nematode-Hotspot-1	27-47	6
	c5-cluster-3	59-79	5			
	c5-cluster-4	84-104	14			
c6	c6-cluster-1	0-17	19	c6-color-Hotspot-1	8-17	6
	c6-cluster-2	24-37	6	c6-Micronaire-Hotspot-4	0-24	4
	c6-cluster-3	53-67	10			
c7	c7-cluster-1	0-25	32	c7-FL-Hotspot-5	0-18	4
	c7-cluster-2	43-59	9	c7-FS-Hotspot-1	0-27	5
	c7-cluster-3	72-91	5	c7-Nematode-Hotspot-2	0-22	9
c8	c8-cluster-1	0-20	6	c8-color-Hotspot-2	20-39	7
	c8-cluster-2	25-39	9			
	c8-cluster-3	107-144	5			
c9	c9-cluster-1	0-20	16			
	c9-cluster-2	29-54	14			
c10	c10-cluster-1	0-20	13	c10-Micronaire-Hotspot-5	0-20	4
	c10-cluster-2	79-112	12			
c11	c11-cluster-1	0-20	26	c11-Nematode-Hotspot-3	0-20	14
c12	c12-cluster-1	0-16	11	c12-FL-Hotspot-6	72-91	6
	c12-cluster-2	18-38	19	c12-FU-Hotspot-1	23-34	5
	c12-cluster-3	67-85	10	c12-Micronaire-Hotspot-6	0-16	4
				c12-Micronaire-Hotspot-7	23-34	4
c13	c13-cluster-1	0-17	6			
	c13-cluster-2	29-44	6			
c14	c14-cluster-1	0-20	30	c14-Micronaire-Hotspot-8	99-118	5
	c14-cluster-2	52-66	10	c-14-Nematode-Hotspot-4	0-15	8
	c14-cluster-3	82-91	7			
	c14-cluster-4	99-122	16			
c15	c15-cluster-1	0-17	8	c15-FE-Hotspot-2	17-39	5
	c15-cluster-2	20-44	24	c15-Leaf-Hotspot-1	17-39	6
	c15-cluster-3	60-68	10	c15-Microanaire-Hotspot-9	23-49	10
c16	c16-cluster-1	0-23	28	c16-Micronaire-Hotspot-10	0-23	8
	c16-cluster-2	30-40	9	c16-VW-Hotspot-1	0-23	5
	c16-cluster-3	50-70	16	c16-VW-Hotspot-2	30-50	7
				c16-VW-Hotspot-3	51-64	4
c17	c17-cluster-1	5-23	12	c17-FU-Hotspot-2	63-78	4
	c17-cluster-2	53-78	13	c17-Micronaire-Hotspot-11	11-27	5
c18	c18-cluster-1	0-19	15	c18-Micronaire-Hotspot-12	28-43	5
	c18-cluster-2	28-43	9			
	c18-cluster-3	53-65	7			
	c18-cluster-4	76-93	8			
c19	c19-cluster-1	0-20	14	c19-FE-Hotspot-3	124-144	4
	c19-cluster-2	32-52	14	c19-Micronaire-Hotspot-13	0-25	4
	c19-cluster-3	62-82	5			
	c19-cluster-4	97-117	5			
	c19-cluster-5	124-144	12			
c20	c20-cluster-1	0-20	7			
	c20-cluster-2	25-45	13			
	c20-cluster-3	117-137	5			
c21	c21-cluster-1	12-32	6	c21-Micronaire-Hotspot-14	60-81	4
	c21-cluster-2	48-64	12			
	c21-cluster-3	70-87	8			
	c21-cluster 4	96-104	5			
c22	c22-cluster-1	0-22	19			
c23	c23-cluster-1	0-22	26	c23-FE-Hotspot-4	0-22	4
	c23-cluster-2	25-35	10	c23-FS-Hotspot-2	77-85	6
	c23-cluster-3	40-70	10	c23-VW-Hotspot-4	0-21	13
	c23-cluster-4	76-87	11	c23-VW-Hotspot-5	26-46	9
	c23-cluster-5	102-119	7			
c24	c24-cluster-1	0-17	13	c24-FS-Hotspot-3	31-51	16
	c24-cluster-2	17-19	6	c24-Micronaire-Hotspot-15	0-19	4
	c24-cluster-3	35-45	17	c24-Micronaire-Hotspot-16	29-35	4
	c24-cluster-4	56-66	5	c24-Micronaire-Hotspot-17	92-110	4
c25	c25-cluster-1	0-21	21	c25-color-Hotspot-3	21-40	4
	c25-cluster-2	40-62	13	c25-color-Hotspot-4	55-75	7
	c25-cluster-3	73-89	17	c25-Micronaire-Hotspot-18	0-21	10
				c25-Micronaire-Hotspot-19	33-65	4
c26	c26-cluster-1	0-22	14	c26-FL-Hotspot-7	0-22	4
	c26-cluster-2	33-51	19	c26-FL-Hotspot-8	45-65	6
				c26-Micronaire-Hotspot-20	28-45	6

**Figure 1:**
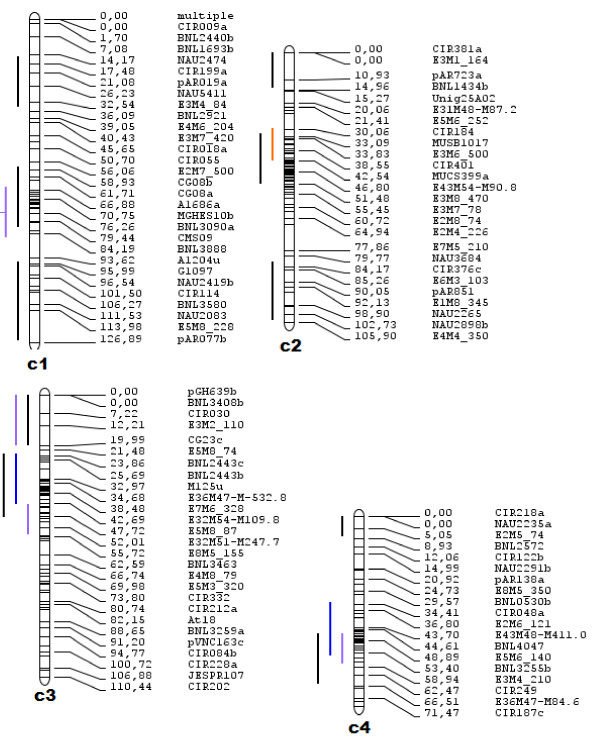
**QTL Clusters and Hotspots.** The figures below represent QTL clusters and hotspots identified. The legend below indicates which trait is assigned to which color.

QTL cluster and hotspot locations on the genome are described in the table below. Locations are given in cM regions which are seen graphically in Figure 1.

### Fiber Quality Trait QTL

#### Fiber strength (FS)

A total of 132 FS QTL were reported over the entire genome except for chromosome 17 (c17) which contained none. Most notably, chromosomes c16, 23, and 24 contained 10, 13, and 26 QTL, respectively. Chromosomes c5, 7, 12, 14, and 18 contained 6, 9, 7, 9, and 6 QTL, respectively. All other chromosomes contained 5 or less FS QTL.

#### Fiber length (FL)

A total of 151 FL QTL were reported over the genome with the exceptions of chromosomes c2 and 22 which contained none. Chromosomes c3, 23, and 26 contained the most QTL with 16, 10, and 14 QTL, respectively. Chromosomes c12 and 19 both contained 9 QTL, while chromosomes c1 and 14 both contained 8 QTL. Chromosome c4, 7, and 25 all contained 7 QTL, while chromosomes 6, 18, and 21 all contained 6 QTL. All other chromosomes contained 5 or less FL QTL.

#### Micronaire

Micronaire QTL were the most frequently identified with 234 QTL spread over the entire genome. Chromosomes c5, 24, and 25 were the most heavily populated with 16, 17, and 20 micronaire QTL, respectively. Chromosome c6 contained 12 QTL, while chromosomes c1, 3, and 12 each contained 11 micronaire QTL. Chromosome c6 contained 10 QTL, while chromosomes c9, 18, and 21 each contained 9 QTL. Chromosomes c10 and 26 both contained 8 QTL. Chromosomes c2, 4, 17, and 23 contained 6, 7, 7, and 6 QTL, respectively. All other chromosomes contained 5 or less QTL.

#### Fiber uniformity (FU)

A total of 91 FU QTL were distributed over the genome with the exception of chromosome c11 which contained none. The majority of QTL were found on chromosomes c12, 17, and 24 which contained 10, 7, and 6 QTL, respectively. All other chromosomes contained 5 or less FU QTL.

#### Fiber elongation (FE)

A total of 118 FE QTL was found across the genome excluding chromosome c11 which contained none. Chromosome c19 contained 12 QTL, while chromosomes c14, 15, 23, and 24 contained 8, 9, 8, and 9 QTL, respectively. Also notable were chromosomes c2 and 20, each of which contained 6 QTL. All other chromosomes contained 5 or less QTL.

#### Color

There were 71 total fiber color related QTL distributed over the genome with the exceptions of chromosomes c4, 12, 13, 20, and 24 which contained none. The most heavily populated chromosomes were chromosomes c6, 8, and 25 which contained 8, 9, and 11 QTL, respectively. Chromosome c9 contained 6 QTL, while all others contained 5 or less.

#### Fiber maturity (FM)

Only 11 FM QTL was identified over the genome with most chromosomes containing either 1 or no QTL. Chromosome c14 contained 2 FM QTL, while chromosomes c3, 5, 9, 10, 12, 16, 18, 24, and 25 all contained only 1 FM QTL.

#### Short lint fibers (SLF)

A total of 2 SLF QTLs were identified and found on chromosomes c18 and 23 each.

#### Weight fitness (WT)

A total of 3 WT QTL were identified on chromosomes c10, 14, and 20.

#### Perimeter

A total of 2 perimeter QTL were identified on chromosomes c10 and 14.

#### Wall thickness

A total of 4 wall thickness QTL were identified to be distributed over 4 chromosomes (each on c3, 5, 9, and 18).

### Yield and Yield Component Trait QTL

#### Boll weight (BW)

A total of 26 BW QTL were identified over the genome in a fairly even distribution. Chromosome c14 contained 4 QTL, while c18 and 22 both contained 3 QTL. Chromosomes c5, 25, and 26 contained 2 QTL each. Chromosomes c1, 2, 4, 11, 12, 15, 16, 21, and 24 all contained 1 QTL, while all others were void of BW QTL.

#### Lint index (LI)

A total of 15 LI QTL was found over the genome with no single chromosome having a notably higher quantity of QTL. Chromosomes c11, 12, 14, and 26 each contained 2 QTL, while chromosomes c4, 5, 7, 9, 10, 22, and 25 contained a single LI QTL.

#### Lint percent (LP)

A total of 25 QTL was found over the genome with no more than 3 QTL on any given chromosome. Chromosomes c7, 11, 23, and 26 all contained 3 LP QTL, while c5, 12, and 16 each contained 2 QTL. Chromosomes c3, 4, 9, 10, 14, 15, 17, and 23 each contained 1 LP QTL, while all other chromosomes contained none.

#### Seedcotton yield (SCY)

A total of 29 SCY QTL were fairly evenly distributed across the genome with no chromosome containing more than 3 SCY QTL. Chromosomes c9, 14, 18, and 26 each contained 3 SCY QTL, while c2, 12, and 25 each contained 2 SCY QTL. Chromosomes c1, 3, 6, 7, 10, 11, 13, 15, 16, 20, and 24 all contained 1 SCY QTL, and all other chromosomes had none.

#### Lint yield (LY)

A total of 23 LY QTL was identified to be distributed over the genome. Chromosomes c1 and 14 each contained 3 LY QTL, while c7, 13, and 18 each contained 2 LY QTL. Chromosomes c3, 5, 6, 9, 12, 15, 16, 20, 22, 24, and 26 all contained only 1 LY QTL.

#### Boll number (BN)

Only 4 BN QTL was reported on 4 different chromosomes (each on c2, 11, 14, and 21).

#### Ratio of log (locule number) to log (boll number) (LB)

Two QTL was identified for this trait one on chromosome c23, and another on c25.

#### Harvest index (HI)

A total of 5 HI QTL were used in the study. Chromosome c2 and 18 contained 2 QTL each, while c14 contained only 1.

### Seed Quality QTL

#### Gossypol

A total of 6 gossypol QTLs were found over the genome with chromosome c19 having 2 QTL. Chromosomes c3, 13, 18, and 22 each contained only 1 gossypol QTL.

#### Seed protein

A total of 29 protein related QTL were distributed over the genome with no chromosome containing more than 3 QTL. Chromosomes c3, 5, and 19 all contained 3 protein QTL, while c2, 6, 12, 15, 20, 21, and 24 each contained 2 QTL. Chromosomes c4, 14, 16, and 22 all contained 1 protein QTL.

#### Seed oil

A total of 16 oil QTLs were used in the study with no more than 3 QTL on any given chromosome. Chromosomes c19 and 21 contained 3 QTL each, while c12 contained 2 QTL. Chromosomes c1, 3, 5, 11, 15, 16, 20, and 24 all contained 1 QTL.

#### Hull percentage (HP)

A total of 2 HP QTL were used in the study and they were found on chromosomes c3 and 14.

#### Embryo protein percentage (EPP)

A total of 2 EPP QTL was used in the study and was found on chromosomes c6 and 15.

#### Number of large fiber seeds (LargenumFS)

Only 1 QTL was used for this trait and it was found on chromosome c12.

#### No fuzz fibers (NOFuzFib)

A total of 2 NoFuzFib QTL was identified both on chromosome 12.

#### Seed weight (SW)

A total of 5 SW QTL were used in the study. Chromosome c9 contained 2 QTL, while c7, 11, and 16 each contained 1 QTL.

#### Seed index (SI)

A total of 10 SI QTL were used in the study. Most notably, chromosome c14 contained 3 QTL, while c3, 7, 17, 22, 23, 24, and 26 contained 1 QTL each.

#### Seed mass (SM)

Only 1 QTL was used for SM and was found on chromosome c2.

### Morphological Trait QTL

#### Fruiting branch number (FB Num)

A total of 4 FB Num QTL on four chromosomes (c5, 11, 14, and 16), each containing 1 QTL were identified.

#### Fruiting branch node (FB Node)

A total of 5 FB Node QTL were identified. Chromosome c16 contained 2 FB Node QTL, while c15, 21, and 25 contained 1 each.

#### Pubescence

A total of 6 pubescence QTLs were identified. Chromosome c6 and 25 each contained 2 pubescence QTLs, while c1 and 23 only contained 1 QTL.

#### Node of first fruiting branch (NFFB)

A total of 6 NFFB QTL were used in the study with chromosome c17 containing 2 QTL. Chromosomes c1, 5, 6, and 11 each contained 1 NFFB QTL.

#### Height of node of first fruiting branch (HNFFB)

Only 1 HNFFB QTL was used in the study and was found on chromosome c17.

#### Leaf morphology (size and shape) (LM)

A total of 25 LM QTL was found across the genome which is associated with leaf size and shape. Most notably chromosome c15 contained 6 QTL, while c17 contained 4 QTL. Chromosomes c6 contained 3 QTL, while c1 and 9 contained 2 QTL each. Chromosomes c2, 3, 4, 10, 12, 18, 22, and 25 all contained 1 QTL.

### Abiotic and Biotic Resistance QTL

#### Osmotic potential (OP)

A total of 5 QTL were found for OP. Chromosome c1 contained 2 OP QTL, while c2, 6, and 25 each contained one.

#### Nematode resistance (Nematode)

A total of 74 nematode resistance related QTLs were used in the study. Of the 74 total QTL, 71 were root-knot nematode and 3 were reniform nematode resistance related QTL. Most notable are chromosomes c7 and 11 which carried 13 and 17 QTL, respectively. Chromosomes c5, 14, and 19 carried 7, 8, and 6 QTL, respectively. Chromosomes c9 and 15 each carried 4 QTL. Chromosomes c20 and 21 each carry 3 QTL, and all other chromosomes carry 2 or less QTL.

#### *Verticillium* wilt resistance (VW)

A total of 63 VW QTL were used in this study. Most notable are chromosomes c16 and 23 which contained 16 and 26 QTL, respectively. Chromosome c5 contained 6 QTL, while c19 contained 3 QTL. Chromosomes c7, 8, 21, 22, and 26 all contained 2 QTL each, while c9 and 24 each contained 1 QTL.

#### Fusarium wilt resistance (Fusarium)

Only 2 Fusarium QTL were used in the study and they were found on chromosomes c2 and 15.

#### *Xanthomonas campestris* pv. *Malvacearum* (Xcm)

A total of 2 Xcm QTL was used in the study and were on chromosomes c5 and 14.

### Physiology Related QTL

#### Leaf chlorophyll

A total of 3 chlorophyll QTLs were used in the study, two of which were on chromosome c2, and one on c14.

### Drought Tolerance QTL Trait

#### Carbon isotope ratio (CIR)

A total of 4 CIR QTL were used in the study. Chromosomes c14, 15, 17, and 25 each contained 1 CIR QTL.

#### Canopy temperature (CT)

Only 1 CT QTL was used in this study and was found on chromosome c6.

The distribution of QTL on individual chromosomes can be analyzed from Table 2. A total of 1,223 QTL were distributed over the entire genome; however, some chromosomes contained high quantities of QTL compared to others. An even distribution of QTL would place about 47 QTL on each chromosome; however, this is not observed based on a Chi-square test (*χ*^2^ =155.7 >37.65 at *P* = 0.05). Chromosomes c14 and 23 each contained 75 QTL, and were the most QTL densely populated chromosomes. They were followed by chromosomes c12, 24, and 25 which contained 61, 72, and 62 QTL, respectively. Also notable are chromosomes c5, 15, and 16 which carried 55, 53, and 57 QTL, respectively. Other chromosomes such as c4, 10, 11, and 20 carried relatively less QTL with 25, 27, 23, and 27 QTL, respectively.

Overall the A subgenome carried 536 QTL, while the D subgenome carried 687 QTL. The Chi-square test (*χ*^2^ =18.72 >3.84 at *P* = 0.05) indicate that QTL were unevenly distributed on the two subgenomes with the D subgenome carrying significantly more QTL identified.

### QTL Clusters and Hotspots

QTL clusters were manually inferred using the chromosomes and QTL positions in Additional file 1: Figure S1. Those clusters were then confirmed using the Biomercator V3 [50] meta-analysis software and are shown in Additional file 1: Figure S2. Clusters were inferred based on regions containing four or more QTL from various traits with a false positive rate of ≤6.25%. QTL hotspots were identified based on only a single trait type where QTL of that trait formed aggregates. Table 3 contains a detailed description of clusters and hotspots identified and their distribution on the genome, and Figure 1 illustrates this table by placing clusters and hotspots on the genome graphically. Table 3 also presents the number of QTL contained in each cluster and hotspot identified. Table 4 is an overview of the number of clusters and hotspots contained on each chromosome. Regions of approximately 20 centimorgans (cM) were taken into account when estimating the presence of a cluster or hotspot. Clusters and hotspots were named according to the chromosome in which they were found. Figure 1 details the positions and intervals of all clusters and hotspots identified.

c1: Chromosome c1 contained 3 QTL clusters carrying 9, 10 and 7 QTL, respectively. The names and spans (i.e. positions) of them are as follows, c1-cluster1 at 14-34cM, c1-cluster-2 at 58-82cM, and c1-cluster-3 at 95-126cM, respectively. The FL hotspot c1-FL-Hotspot-1 carrying 5 QTL was located at 66-86cM.

c2: Chromosome c2 contained 3 clusters and 1 FE hotspot. The clusters c2-cluster1, c2-cluster-2 and c2-cluster-3 were found at 0-14cM, 32-52cM and 82-105cM, and carried 5, 13 and 5 QTL, respectively. The one hotspot identified as c2-FE-Hotspot-1 was located at 30-43cM, and carried 4 QTL. It should be noted that the hotspot identified coincided with the position of the second cluster found.

c3: Chromosome c3 contained 2 clusters and 3 hotspots. The clusters c3-cluster1 and c3-cluster-2 had spans (i.e., positions) of 0-20cM and 23-48cM, and carried 15 and 23 QTL, respectively. The first two hotspots identified were FL hotspots, c3-FL-Hotspot-2 and c3-FL-Hotspot-3, had spans of 0-20cM and 43-55cM, and carried 7 and 5 QTL, respectively. The third hotspot was a micronaire hotspot with 5 QTL named c3-Micronaire-Hotspot-1 and ranged from 23-43cM. Again, all hotspots coincided with the identified clusters on the chromosome.

c4: Chromosome c4 had two clusters, c4-cluster-1 and c4-cluster-2 and ranged from 0-8cM and 46-66cM, and carried 7 and 10 QTL, respectively. The two hotspots identified were found around the region of the second cluster, one was a FL hotspot while the other was a micronaire hotspot. The hotspot c4-FL-Hotspot-4 ranged from 46-58cM and carried 5 QTL, while the micronaire hotspot c4-Micronaire-Hotspot-2 ranged from 34-55cM and carried 4 QTL.

c5: Chromosome c5 contained 4 clusters and 2 hotspots. The clusters c5-cluster-1, c5-cluster-2, c5-cluster-3 and c5-cluster-4 were identified at 0-20cM, 22-42cM, 59-79cM and 84-104cM, and carried 15, 16, 5, and 14 QTL, respectively. The micronaire hotspot c5-Micronaire-Hotspot-3 was identified at 0-20cM and carried 5 QTL. The nematode resistance related hotspot c5-Nematode-Hotspot-1 was identified at 27-47cM and carried 6 QTL.

c6: Chromosome c6 contained 3 clusters and 2 hotspots. The clusters c6-cluster-1, c6-cluster-2 and c6-cluster-3 were identified at 0-17cM, 24-37cM and 53-67cM, and carried 19, 6 and 10 QTL, respectively. The first hotspot pertained to color c6-color-Hotspot-1 and was found within the first cluster at 8-17cM, and carried 6 QTL. The other hotspot pertaining to micronaire fell within the range of the second cluster. The micronaire hotspot c6-Micronaire-Hotspot-4 was found at 0-24cM and carried 4 QTL.

c7: Chromosome c7 contained 3 clusters and 3 hotspots. The clusters c7-cluster-1, c7-cluster-2 and c7-cluster-3 were identified at 0-25cM, 43-59cM and 72-91cM, and carried 32, 9 and 5 QTL, respectively. The three hotspots identified fell into the range of the first cluster and were FL, FS, and nematode resistance related hotspots, respectively. The first hotspot c7-FL-Hotspot-5 and the second c7-FS-Hotspot-1 overlapped at 0-18cM and 0-27cM, indicating a possible fiber quality hotspot cluster in the region. The FL hotspot carried 4 QTL, while the FS hotspot carried 5 QTL. The nematode resistance related QTL hotspot c7-Nematode-Hotspot-2 overlapped the first cluster at 0-22cM and carried 9 QTL.

c8: Chromosome c8 contained 3 clusters, c8-cluster-1, c8-cluster-2 and c8-cluster-3, which ranged from 0-20cM, 25-39cM and 107-144cM, and carried 6, 9 and 5 QTL, respectively. One hotspot was identified as c8-color-Hotspot-2, carrying 7 QTL at 20-39cM and fell into the range of the second cluster.

c9: Chromosome c9 only contained two clusters, c9-cluster-1 and c9-cluster-2, which had ranges of 0-20cM and 29-54cM, and carried 16 and 14 QTL, respectively. But no hotspots were identified.

c10: Chromosome c10 contained 2 clusters and 1 hotspot. The two clusters c10-cluster-1 and c10-cluster-2 had ranges of 0-20cM and 79-112cM, and carried 13 and 12 QTL, respectively. The micronaire hotspot c10-Micronaire-Hotspot-5 was identified at 0-20cM, carried 4 QTL, and fell into the region of the first cluster.

c11: Chromosome c11 contained 1 cluster and 1 hotspot. Cluster c11-cluster-1 was identified at 0-20cM and carried 26 QTL. The hotspot c11-Nematode-Hotspot-3 overlapped the cluster at 0-20cM and carried 14 QTL.

c12: Chromosome c12 contained 3 clusters and 4 hotspots. The clusters named c12-cluster-1, c12-cluster-2 and c12-cluster-3 were identified at 0-16cM, 18-38cM and 67-85cM, and carried 11, 19 and 10 QTL, respectively. The first hotspot c12-FL-Hotspot-6 was identified at 72-91cM, and carried 6 QTL. The second hotspot c12-FU-Hotspot-1 ranged from 23-34cM and carried 5 QTL. The last two micronaire hotspots c12-Micronaire-Hotspot-6 and c12-Micronaire-Hotspot-6 were identified at 0-16 and 23-34cM, respectively, and carried 4 QTL each. The second micronaire hotspot and the FU hotspot overlapped perfectly, indicating another possible fiber quality hotspot cluster region.

c13: Chromosome c13 only contained 2 clusters and no hotspots. The clusters c13-cluster-1 and c13-cluster-2 ranged from 0-17cM and 29-44cM, and both carried 6 QTL, respectively.

c14: Chromosome c14 contained 4 clusters and 2 hotspots pertaining to micronaire and nematode resistance related QTL. The four clusters c14-cluster-1, c14-cluster-2, c14-cluster-3 and c14-cluster-4 were identified at 0-20cM, 52-66cM, 82-91cM and 99-122cM, and carried 30, 10, 7, and 16 QTL, respectively. The micronaire hotspot overlaps with the fourth cluster at 99-118cM and was designated c14-Micronaire-Hotspot-8, and carried 5 QTL. The nematode resistance related hotspot overlaps the first cluster at 0-15cM and carried 8 QTL.

c15: Chromosome c15 contained 3 clusters and 3 hotspots. The clusters c15-cluster-1, c15-cluster-2 and c15-cluster-3 were identified at 0-17cM, 20-44cM and 60-68cM, and carried 8, 24 and 10 QTL, respectively. The three hotspots were pertaining to FE, leaf morphology, and micronaire. The FE hotspot, c15-FE-Hotspot-2 had a range which overlapped perfectly with c15-Leaf-Hotspot-1 and both were identified at 17-39cM. The FE hotspot carried 5 QTL, while the leaf morphology hotspot carried 6 QTL. There was also partial overlap of the third hotspot c15-Micronaire-Hotspot-9 carrying 10 QTL at 23-49cM, indicating another possible fiber hotspot cluster region.

c16: Chromosome 16 contained 3 clusters and 4 hotspots. The clusters c16-cluster-1, c16-cluster-2 and c16-cluster-3 were identified at 0-23cM, 30-40cM and 50-70cM, and carried 28, 9, and 16 QTL, respectively. The first hotspot c16-Micronaire-Hotspot-10 had a range of 0-23cM and carried 8 QTL. The other three hotspots were all for *Verticillium* wilt (VW) resistance hotspots. The first VW hotspot c16-VW-Hotspot-1 overlaps perfectly with the micronaire hotspot at 0-23cM and carried 5 QTL. The hotspots c16-VW-Hotspot-2 and c16-VW-Hotspot-3 were identified at 30-50cM and 51-64cM, and carried 7 and 4 QTL, respectively.

c17: Chromosome c17 contained 2 clusters and 2 hotspots. The clusters c17-cluster-1 and c17-cluster-2 had ranges of 5-23cM and 53-78cM, and carried 12 and 13 QTL, respectively. The first hotspot c17-FU-Hotspot-2 fell within the range of the second cluster at 63-78cM and carried 4 QTL, whereas the second c17-Microanire-Hotspot-11 overlapped the first cluster with a range of 11-27cM and carried 5 QTL.

c18: Chromosome c18 contained 4 clusters and 1 micronaire hotspot. The clusters c18-cluster-1, c18-cluster-2, c18-cluster-3 and c18-cluster-4 had ranges of 0-19cM, 28-43cM, 53-65cM and 76-93cM, and carried 15, 9, 7 and 8 QTL, respectively. The one micronaire hotspot c18-Micronaire-12 was identified at 28-43cM which was associated with the second cluster and carried 5 QTL.

c19: Chromosome c19 contained 5 clusters and 2 hotspots. The clusters c19-cluster-1, c19-cluster-2, c19-cluster-3, c19-cluster-4 and c19-cluster-5 were identified at 0-20cM, 32-52cM, 62-82cM, 97-117cM and 124-144cM, and carried 14, 14, 5, 5 and 12 QTL, respectively. The hotspot c19-FE-Hotspot-3 had a range of 124-144cM and carried 4 QTL. The micronaire hotspot c19-Micronaire-Hotspot-13 had a range of 0-25cM and carried 4 QTL.

c20: Chromosome c20 contained 3 clusters, c20-cluster-1, c20-cluster-2 and c20-cluster-3, which were found at 0-20cM, 25-45cM and 117-137cM, and carried 7, 13 and 5 QTL respectively. No hotspots were found on this chromosome.

c21: Chromosome c21 had 4 clusters and 1 micronaire hotspot. The clusters c21-cluster-1, c21-cluster-2, c21-cluster-3 and c21-cluster-4 were identified at 12-32cM, 48-64cM, 70-87cM and 96-104cM, and carried 6, 12, 8 and 5 QTL, respectively. The one hotspot c21-Micronaire-Hotspot-14 coincided between the second and third cluster at 60-81cM due to long confidence intervals of some micronaire QTL, and contained at total of 4 QTL.

c22: Chromosome c22 had 1 cluster c22-cluster-1 and was identified at 0-22cM, and carried 19 QTL. No hotspots were identified on this chromosome.

c23: Chromosome c23 had 5 clusters and 4 hotspots. The clusters c23-cluster-1, c23-cluster-2, c23-cluster-3, c23-cluster-4 and c23-cluster-5 were identified at 0-22cM, 26-35cM, 40-70cM, 76-87cM and 102-119cM, and carried 26, 10, 10, 11 and 7 QTL, respectively. The hotspots c23-FE-Hotspot-4 carrying 4 QTL and c23-VW-Hotspot-4 carrying 14 QTL overlapped at 0-22cM and 0-21cM, respectively. The hotspot c23-FS-Hotspot-2 was identified at 77-85cM and carried 6 QTL. The other VW hotspot c23-VW-Hotspot-5 was identified at 26-46cM and carried 9 QTL.

c24: Chromosome c24 contained 4 clusters and 4 hotspots. The clusters c24-cluster-1, c24-cluster-2, c24-cluster-3 and c24-cluster-4 were found at 0-17cM, 17-19cM, 35-45cM and 56-66cM, and carried 13, 6, 17, and 5 QTL, respectively. The clusters on this chromosome were slightly tighter with smaller ranges as QTL were tightly aggregated into smaller regions of the genome. The first hotspot c24-FS-Hotspot-3 was found at 31-51cM and carried 16 QTL. The other three micronaire hotspots c24-Micronaire-15, c24-Micronaire-16 and c24-Microanire-17 were found at 0-19cM, 29-35cM and 92-110cM, and carried 4 QTL each. The second micronaire hotspot and the FS hotspot overlapped, indicating a possible aggregation of fiber quality QTL hotspots.

c25: Chromosome c25 had 3 clusters and 4 hotspots, some of which overlapped each other. The clusters c25-cluster-1, c25-cluster-2 and c25-cluster-3 were identified at 0-21cM, 40-62cM and 73-89cM, and carried 21, 13 and 17 QTL, respectively. The first two hotspots pertaining to fiber color, c25-color-Hotspot-3, and c25-color-Hotspot-4, were identified at 21-40cM and 55-75cM and carried 4 and 7 QTL, respectively. The other two micronaire hotspots, c25-Micronaire-Hotspot-18, and c25-Micronaire-Hotspot-19, were identified at 0-21cM and 33-65cM, and carried 10 and 4 QTL, respectively. The second fiber color hotspot and the second micronaire hotspot overlapped partially and may constitute another fiber quality QTL hotspot cluster region.

c26: Chromosome c26 contained 2 clusters and 3 hotspots. The clusters c26-cluster-1 and c26-cluster-2 had ranges of 0-22cM and 33-51cM, and carried 14 and 19 QTL, respectively. The first two hotspots pertaining to fiber length (FL) were c26-FL-Hotspot-7, and c26-FL-Hotspot-8 were identified at 0-22cM and 45-65cM, and carried 4 and 6 QTL, respectively. The micronaire hotspot c26-Micronaire-Hotspot-20 at 28-45cM carried 6 QTL.

**Table 4:** Summary of QTL clusters and hotspots

**Chromosome**	**Number of clusters**	**Number of hotspots**
**c1**	3	1
**c2**	3	1
**c3**	2	3
**c4**	2	2
**c5**	4	2
**c6**	3	2
**c7**	3	3
**c8**	3	1
**c9**	2	0
**c10**	2	1
**c11**	1	1
**c12**	3	4
**c13**	2	0
**c14**	4	2
**c15**	3	3
**c16**	3	4
**c17**	2	2
**c18**	4	1
**c19**	5	2
**c20**	3	0
**c21**	4	1
**c22**	1	0
**c23**	5	4
**c24**	4	4
**c25**	3	4
**c26**	2	3

The table below shows the number of clusters and hotspots associated with each chromosome.

Taken together, 76 QTL clusters were cumulatively identified and each chromosome contained at least 1 cluster. Chromosomes c19 and 23 had the most clusters with 5 clusters each, while chromosomes c11 and 22 only contained 1 cluster each. Cumulatively 51 QTL hotspots were identified with some chromosomes containing no hotspots, while others contained three or more hotspots. Chromosomes c12, 16, 23, 24 and 25 carried 4 QTL hotspots each, while c9, 13, 20 and 22 contained no hotspots. A total of 3 fiber strength (FS) QTL hotspots were identified on c7, 23, and 24, respectively. In total 8 fiber length (FL) QTL hotspots were identified. Chromosome c1, 4, 7 and 13 each contained 1 FL QTL hotspot, while c3 and 26 each contained 2 QTL FL hotspots. A total of 4 fiber elongation (FE) QTL hotspots were identified on chromosomes c2, 15, 19, and 23 each. A total of 2 fiber uniformity (FU) QTL hotspots were identified, one was on c12 and the other on c17. Micronaire QTL hotspots were more abundant than others identified with a total of 20 hotspots. Chromosomes c3, 4, 5, 6, 10, 14, 15, 16, 17, 18, 19, 21, and 26 each contained 1 micronaire QTL hotspot; c12 and 25 each contained 2 hotspots; and c24 contained 3 micronaire QTL hotspots. A total of 4 fiber color hotspots were identified. Chromosomes c6 and 8 each contained 1 fiber color QTL hotspot, while c25 contained 2 QTL hotspots. A total of 1 leaf morphology QTL hotspot was identified on chromosome c15. A total of 5 *Verticillium* wilt (VW) resistance QTL hotspots were identified. Chromosome c16 contained 3 VW resistance QTL hotspots, while c23 contained 2 VW resistance QTL hotspots. A total of 4 nematode resistance related QTL hotspots were identified in that chromosome c6, 7, 11, and 14 each carried one hotspot.

Distribution of clusters and hotspots were further compared between respective homoelogous chromosomes from the A and D subgenomes. Eight homologous chromosome pairs, i.e., c1(A1) and c15(D1), c2(A2) and c14(D2), c3(A3) and c17(D3), c4(A4) and c22(D4), c8(A8) and c24(D8), c9(A9) and c23(D9), c11(A11) and c21(D11), and c13(A13) and c18(D13) did not share any remarkable similarities in their number or placement of clusters or hotspots. Two homoelogous chromosome pairs c7(A7) and c16(D7), and c10(A10) and c20(D10), both carried clusters at the same region (0-30cM and 0-20cm, respectively). The c7(A7) and c16(D7) pair each also carried a hotspot but they were of different trait types. Interestingly, three homoelogous chromosome pairs c5(A5) and c19(D5) at 0-20cM, c6(A6) and c25(D6), and c12(A12) and c26(D12) at 25-45cM, both contained or shared a micronaire hotspot at similar regions (i.e., approximately 0-20cM). Chromosomes c6(A6) and c25(D6) each also carried a color hotspot while c12(A12) and c26(D12) each had a fiber length hotspot; however, the hotspots did not coincide on similar regions of the homeologous chromosomes. Therefore, overall, the 13 pairs of homeologous chromosomes from the A and D subgenomes do not have remarkable similarities of QTL clusters or hotspots in number or placement on the genome.

Chromosomes containing two or more QTL hotspots always contained at least 2 different trait QTL hotspot types. All hotspots overlapped with clusters in the same region. Some chromosomes containing more than one hotspot contained regions where hotspots overlapped forming hotspot clusters. For example, chromosome c4 contained an overlap between a FL and a micronaire QTL hotspot. Chromosome c6 contained an overlapping fiber color QTL hotspot and a micronaire QTL hotspot. Chromosome c7 contained an overlap between a FS, nematode resistance, and a FL QTL hotspot. Chromosome c12 contained an overlapping FU and micronaire QTL hotspots. Notably, chromosome c15 contained a FE, leaf morphology, and a micronaire QTL hotspot, all of which overlapped in the same region. Chromosome c16 contained an overlap between a VW resistance QTL hotspot and a micronaire QTL hotspot. Chromosome c23 contained two overlapping QTL hotspots for FE and VW resistance. Chromosome c24 contained an FS and a micronaire QTL hotspot which overlapped forming a fiber quality hotspot cluster. Chromosome c25 contained a fiber color and a micronaire QTL hotspot which overlapped forming another fiber quality hotspot cluster.

## Discussion

The 1,223 QTL distributed over the *Gossypium* genome in this study revealed the presence of QTL clusters and specific trait QTL hotspots. Certain regions of the cotton chromosomes were more densely populated with QTL than other regions, implying the existence of QTL clusters. Hotspots for fiber quality, *Verticillium* resistance, nematode related resistance and leaf morphology were identified. Regions which contained clusters often contained hotspots, and in some cases hotspots overlapped. This data could potentially be used to identify new QTL in defined regions by clusters rather than attempting to scan the entire genome for QTL.

The significance of this study is the discovery of QTL clusters and hotspots, and reaffirmation of previous meta-analysis studies, which are of immediate value to breeders. The comprehensive cluster and hotspot map produced in this study will allow breeding programs to focus their efforts on regions of the genome containing the most QTL of interest. In addition, breeding programs may utilize the confidence interval data to only focus on QTL within narrow regions of the genome, as opposed to QTL containing broad confidence intervals. Hotspot clusters containing two or more hotspots will be of immediate interest to breeding programs as they contain multiple QTL of interest. In addition, this study is a much needed update of previous meta-analysis studies in *Gossypium* that is less comprehensive and up to date [3,4]. Previous meta-analyses have shown that QTL overlap to form clusters and that certain regions of the genome contain more QTL than others [3,4]. Therefore it is possible that novel QTL may be found within the clusters identified by this study.

In this study, data was collected from 42 different publications using a variety of population types (see Table 1), *Gossypium* species, environments, and QTL traits. Each study varied in their reporting of QTL in terms of confidence intervals used and LOD scores at which QTL were declared. The Biomercator V3 software requires confidence intervals (CI) in the QTL report; however, some publications did not include them. For those publications CI were estimated based on flanking marker positions of the QTL placed on the chromosome. Only QTL with current markers found in the cotton marker database and did not have multiple locations on a linkage groups were used in this study. When exact QTL positions were unknown and only flanking markers positions were given the average distance between markers was used to place the QTL on the chromosome. Also when exact confidence intervals were not provided, the flaking marker positions were used as confidence intervals. Each publication varied in terms of the average length of CI with some being significantly longer than others. The identification of clusters and hotspots are more problematic using QTL with wide CI. Not all QTL from every study was included in the analysis for the reasons described above to preserve accuracy in the study. Some studies identified QTL on A and D linkage groups and used flanking markers which did not coincide with markers known based on the Cotton Marker Database (CMD). For those QTL it was not possible to place them on the consensus map and they were therefore excluded from the study. QTL which were outside the range of the consensus map provided by the CMD were excluded from the study. Clusters were estimated within regions of approximately 20cM allowing for the possibility of QTL with wide confidence intervals to be possibly placed anywhere in that region. The Biomercator V3 software [50] also required LOD scores and R^2 (phenotypic variation explained by the QTL) values for all QTL in the study. Both the LOD scores and R^2 values varied wildly between studies; however, this data was irrelevant to the QTL placement on the chromosomes. QTL placement and inference of clusters were based only on the physical placement of the QTL in genetic distance (cM) given by each study, and the CI which was estimated by flanking markers if not given in the study.

The majority of the QTL data in this analysis was taken from fiber quality QTL analysis and not studies pertaining to yield traits or resistance QTL to biotic and abiotic stresses. Thus, the large QTL meta-analysis studies done in *Gossypium* have focused only on fiber quality traits [3,4]. This study pools together not only large scale fiber quality QTL studies but also yield, seed quality, leaf morphology, and resistance studies. The majority of hotspots were fiber quality related; however, five resistance, and one leaf morphology hotspot were identified. The existence of hotspots pertaining to multiple traits coexisting in the same region of a chromosome or hotspot clusters is beneficial to breeders, and some have been identified in this study. The impact of this study is the identification of fiber quality, yield, seed, leaf morphology, and resistance hotspots, and clusters containing various mixtures of all traits. The identification of clusters and hotspots will be useful in marker-assisted selection since the markers delineating these regions can be chosen for selecting the traits of interests in cotton breeding. For example, many of the QTL hotspots identified in this study contained hotspot cluster regions containing two or more hotspots pertaining to different traits. In this study, such regions often pertained to fiber quality related QTL hotspots. Breeding programs focused on fiber quality traits can focus on hotspot clustering regions and select for the flanking markers around the region. Most of the QTL clusters had hotspots associated with them, indicating that if new QTL are to be discovered they may be found around regions of known clusters. Marker assisted selection programs can utilize clusters found in this study to find novel QTL and possibly novel hotspots within the regions. This study identified regions of importance to marker assisted breeding programs with clusters and hotspots, and identified chromosomes which have the most QTL, clusters, and hotspots for future breeding programs.

To make the results publicly accessible, a database will be established. Updates will be also made regularly when new studies have been published. Users can access the information based on their needs or interests per trait or chromosome.

## Conclusion

QTL clusters and hotspots were inferred and identified using the positions and distribution of QTL along the *Gossypium* genome. The presence of QTL clusters and hotspots indicate that genes pertaining to certain traits are more heavily concentrated in certain regions of the genome than others. Since multiple *Gossypium* species were used in the various publications it confirms that QTL clusters and hotspots are consistent throughout the genus. The study found clusters on every chromosome, but hotspots pertaining to specific traits are present only on some chromosomes. The locations of these clusters and hotspots will be beneficial for marker assisted selection and breeding programs focused on fiber quality, seed quality, disease resistance, leaf morphology, and other yield related traits. Fiber quality hotspots dominated this study, but five disease resistance hotspots, four pest resistance hotspots, and one leaf morphology hotspot were detected. The chromosomal locations of these clusters can be used as a starting point to identify new QTL using consensus marker and meta-analysis data. This may be useful for future QTL analysis to map regions of the genome with high phenotypic impact for various traits. To date this is the most comprehensive QTL meta-analysis study done with Gossypium as it utilizes past meta-analyses and current publications to identify novel clusters and hotspots previously not described.

## Methods

Table 1 contains a summary of the QTL and publications used in this study. This study incorporated reported QTL from other studies onto a consensus map created by the Cotton Marker Database (CMD) [51] based on *G. hirsutum* x *G. barbadense*. The traits reported and used in this study are the following:

Fiber strength (FS): Fiber strength is described as the necessary force to break a beard of fibers that are clamped between two sets of jaws and is measured in grams per denier [51].

Fiber length (FL): Fiber length is the description of the average length of the longest half of the fibers (upper half) and can be described as the upper half mean length measured in millimeters [51].

Micronaire (Micro): Micronaire is a measurement of fiber fineness and maturity. A mass of 2,34g of fibers is compressed into a space of known volume and air permeability and the compression of the sample is recorded as a micronaire value [51].

Fiber uniformity (FU): Fiber uniformity is an expression of the ratio of upper-half fiber length and the mean length and is denoted as a percentage [51].

Fiber elongation (FE): Fiber elongation is a measurement of deformation under tensile stress. The measurement is a percentage change in length based on the length of the sample prior to tensile stress [51].

Fiber color (Color): Fiber color is sometimes called reflectance or yellowness and is a measurement of the yellow tint in the fibers [52].

Fiber maturity (FM): Fiber maturity is described as the extent of development in the secondary walls [51].

Fiber perimeter (Perimeter): Fiber perimeter is a diverse trait which directly relates to fiber strength, length, and micronaire and is considered a fiber quality trait [53].

Weight fitness (WT): WT is a fiber quality trait relating to maturity which is measured by an arealometer, a device which measures compressional change in a known unit volume space with a known weighted sample [42].

Wall thickness (Wall Thick): Wall thickness is described as the thickness of the fiber in the secondary wall [51].

Short lint fibers (SLF): The content of short lint fibers is defined as percent by weight of fibers of 12.7 mm or less [52].

Harvest index (HI): The harvest index is a ratio by weight of seedcotton to dry matter produced by a cotton plant [53].

Boll weight (BW): Boll weight is the average weight in grams of a boll [51].

Lint index (LI): Lint index is described as the lint obtained from 100 seeds and is measured in grams [9].

Lint percentage (LP): Lint percent is a ratio by weight of lint to seedcotton [51].

Seed cotton yield (SCY): SCY is an expression of seedcotton weight in kg per harvested unit area (ha) [51].

Lint yield (LY): Lint yield is an expression of lint weight in kg per harvested unit area (ha) [51].

Boll number (BN): Boll number is described as the number of bolls per plant [51].

Ratio of log (locule number) to log (boll number) (LB): LB is a calculation of the ratio of the log of locule number to the log of the number of bolls present [18].

Seed gossypol content (Gossypol): Gossypol is expressed as a percentage of gossypol in weight found in the cotton seed [54].

Seed protein content (Protein): Protein is expressed as the percentage of protein in weight found in the cotton seed [44].

Seed oil content (Oil): Oil is expressed as the percentage of oil in weight found in the cotton seed [44].

Hull percentage (HP): HP is an expression of the percentage of seed which comprises the hull of the embryo [9].

Embryonic protein percentage (EPP): EPP is a percentage of protein in weight present in the embryo [9].

Number of large fiber seeds (LargenumFS): Expressed as the number of large fiber seeds present. The trait relates to seed mass, with larger seeds having greater fitness than smaller seeds [5].

No fuzz fibers (NOFuzFib): No fuzz fibers is a trait described by seeds which only have lint fibers and no fuzz fibers [55].

Seed weight (SW): Seed weight is measured in grams [51].

Seed index (SI): Seed index is described as the weight of 100 seeds in grams [51].

Seed mass (SM): Seed mass is expressed as the seed mass in grams per unit area [3].

Number of fruiting branches (FB Num): Described as the number of fruiting branches per plant [14].

Number of fruiting branch nodes (FB Node): Described as the number of fruiting nodes per plant [14].

Node of first fruiting branch (NFFB): Used as a test of plant maturity and development by the examination of the maintem node (counted from the first true leaf) of the first fruiting branch [20].

Height of node of first fruiting branch (HNFFB): A measurement of height of the first fruiting branch which relates to maturity and development traits [20].

Leaf morphology (Leaf Morph): Leaf morphology traits are described as differences between plants in leaf size, shape, and the number of lobes in each leaf [52].

Osmotic potential (OP): The osmotic potential is described as the plant’s ability to adjust to osmotic differences via the active accumulation of solutes in response to a water deficit. This trait pertains to drought tolerance [27].

Nematode related resistance (Nematode Related): Nematode resistance related traits pertain to both reniform and root-knot nematodes. QTL for this trait are classified based on the number of eggs per gram root per plant, and the distribution of root galling index [35].

Fusarium wilt resistance (Fusarium): Fusarium resistance is a measurement of the plant’s survival and resistance (disease severity rating) of infection by the inoculum [39].

Bacterial blight resistance (Xcm): Refers to traits which allow the plant to resist infection after being inoculated with Xcm [52].

Verticillium wilt (VW): Refers to traits (percentage of plants infected or disease severity rating) which allow the plant to resist infection after being inoculated with VW fungus [37].

Chlorophyll content (Chlorophyll): A measurement of the amount of chlorophyll present in the leaves. This trait describes QTL which appear to have a direct impact on chlorophyll content [27].

Carbon isotope ratio (CIR): A ratio of the carbon isotopes present which relates directly to a plant’s ability to use water efficiently. CIR is a drought tolerance QTL trait [27].

Canopy temperature (CT): This trait is a measurement of canopy temperature and the amount of abiotic stress associated with the temperature [20]. CT is a drought tolerance related QTL trait [52].

Biomercator V3 [50] is capable of incorporating map files with QTL data files and displaying the results in a graphical representation. Map files consist of marker names along with the distance of the marker from the previous marker. The software then constructs the map based on adding the marker distances and displays the map along with markers with the appropriate distances between them. The QTL files consist of the map name, QTL name, chromosome number, trait, LOD score, phenotypic variance explained (R^2), mapping method used by the publication, position of the QTL, and the confidence interval.

The Biomercator V3 meta-analysis algorithm works by using a maximum likelihood method to calculate the most likely QTL distribution [56]. The CI, R^2, LOD scores, and positions of each QTL are assessed when calculating the existence of a cluster [56]. The algorithm assumes that each input QTL is not a false positive [56]. The algorithm then computes each possible model using the input QTLs and determines the most likely model [56]. Meta-analysis is a two-step process using Biomercator V3 [50]. First the linkage group on a specific chromosome is selected along with QTL of choice. During cluster analysis all QTL for a specific chromosome were included. The default kMax setting of 10 was used which in the second step allowed the software to calculate up to 10 possible clusters [50]. No chromosome in the study contained more than 5 clusters, so in the second step the program was instructed to find the best number of clusters appropriate for each chromosome based on the manual inference data.

Mapping methods varied between studies using CIM, MQM, and ICIM; however, this did not affect the Biomercator software’s ability to place all QTL on the consensus map. Different QTL trait types are represented using different colors on chromosome maps. Each QTL is represented by a small horizontal line and a perpendicular vertical line. The horizontal line indicates the position in cM on the chromosome, and the vertical line represents the confidence interval of the QTL position on the map. The software is capable of calculating possible meta-clusters of QTL based on the number and position of QTL in a given region of the chromosome.

Meta-analysis was performed on each chromosome manually and using the software. Using manual inference both clusters and hotspots pertaining to specific traits were declared. These clusters and hotspots were then projected on the genome using Biomercator V3 software [50]. The same consensus map used for the Biomercator portion of the study was used and cluster and hotspot intervals were estimated based on marker positions on the map. The meta-analysis software used by Biomercator requires an input between 1 and 10 to display the “best meta-regions”. For this reason the number of clusters estimated by the manual inference portion was used as input to the software. For example when the manual inference detected 2 clusters the software was configured to find the “best” 2 clusters. If the software was configured to find the “best” 3 or 4 clusters it often declared false positives, declaring a cluster when only 2 or 3 QTL were present. Hotspots were declared manually by removing all QTL trait types except for one to detect dense regions of that QTL type. Both clusters and hotspots were declared within approximately 20cM regions, meaning if a multiple QTL were detected between 0 and 20cM one cluster or hotspot was declared. This method of declaring clusters and hotspots with 20cM regions is based on the observation that large aggregates of QTL usually existed within a region about that size.

## Competing interests

The authors declare that they have no competing interests.

## Authors’ contributions

JFZ conceived the project and contributed to the editing and writing of the manuscript. JIS carried out the project and drafted the manuscript. ZXL and XLZ provided editorial advice of the manuscript. MZS participated in the project. All authors read and approved the final manuscript.

## Supplementary Material

Additional file 1: Figure S1 QTLs and their distribution on the genome. **Figure S2**. Meta-analysis performed by Biomercator V3. **Figure S3**. Fiber strength hotspots present. **Figure S4**. Fiber length hotspots. **Figure S5**. Fiber uniformity hotspots. **Figure S6**. Fiber elongation hotspots. **Figure S7**. Micronaire hotspots. **Figure S8**. Color hotspots. **Figure S9**. Leaf morphology hotspots. **Figure S10**. Verticillium wilt resistance (VW) hotspots. **Figure S11**. Nematode Resistance hotspots. Click here for file
