# Supplementary material for: A comprehensive meta QTL analysis for fiber quality, yield, yield related and morphological traits, drought tolerance, and disease resistance in tetraploid cotton
Source: BMC Genomics. 2013 Nov 11;14:776. doi: 10.1186/1471-2164-14-776 (PMC3830114; doi:10.1186/1471-2164-14-776)

**Additional file**

**Figure S1 - QTLs and their distribution on the genome**

Figure S1: The figures below are all 1223 QTL from the 42 studies [4, 9-49] distributed over the entire *Gossypium* genome. Each vertical line represents a confidence interval and each horizontal line represents the placement of a QTL given by a particular study. The marker names and cM location on the chromosome appears on the right side of the chromosome in black. Below chromosome 26 is a color coded legend which identifies each QTL trait type.

**
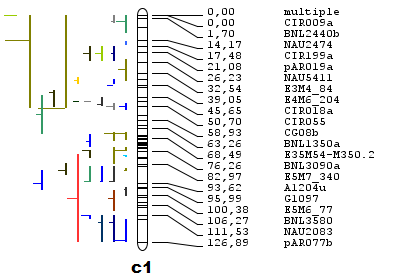

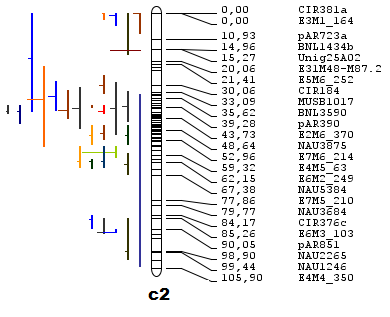

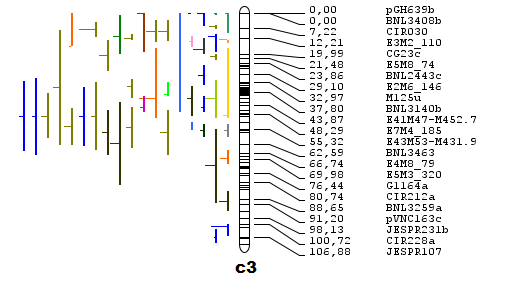

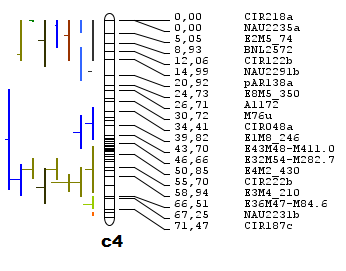

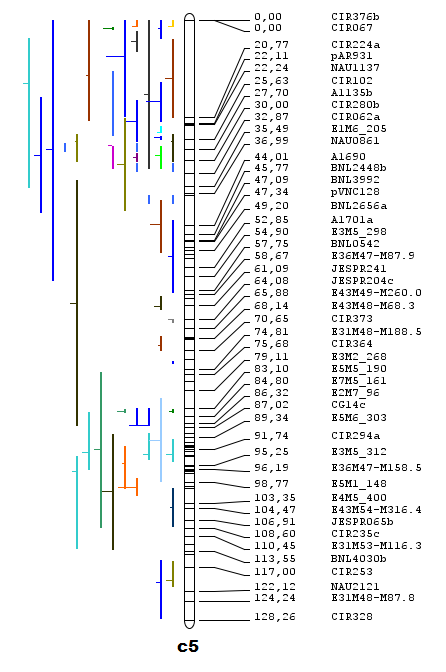

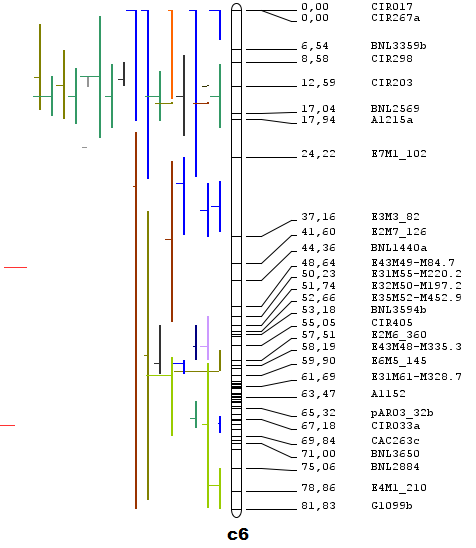

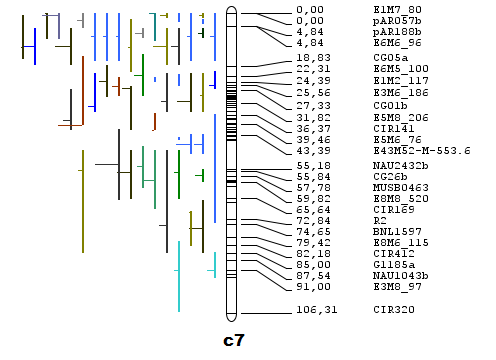

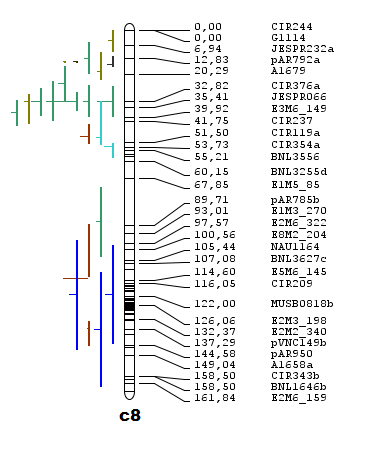

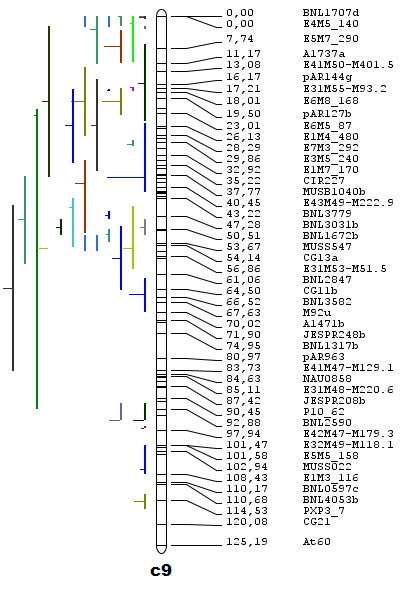

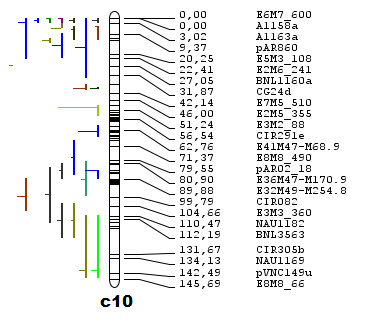

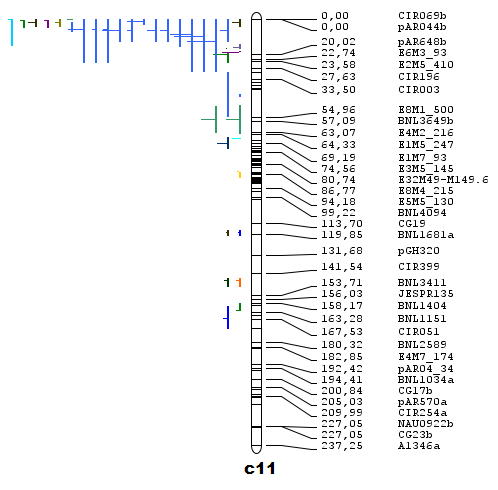

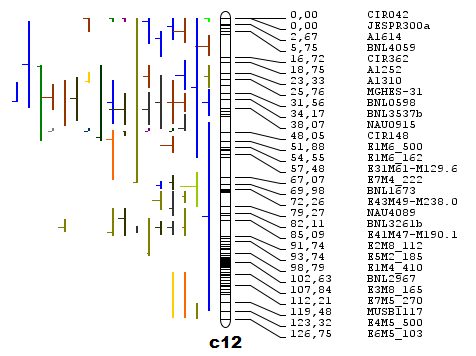

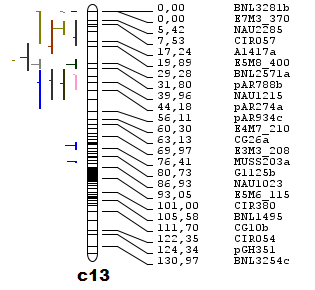

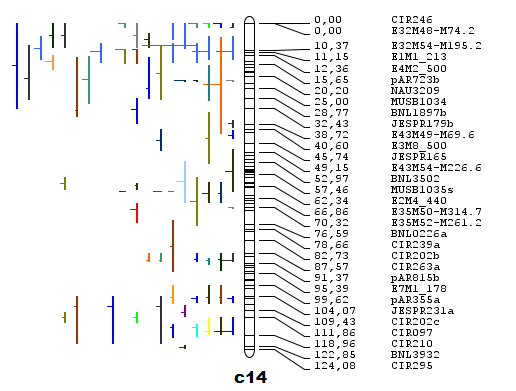

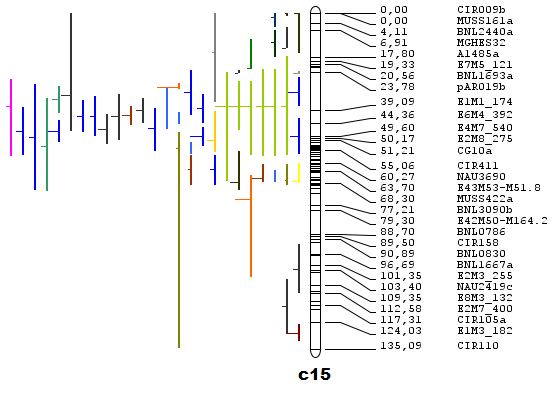

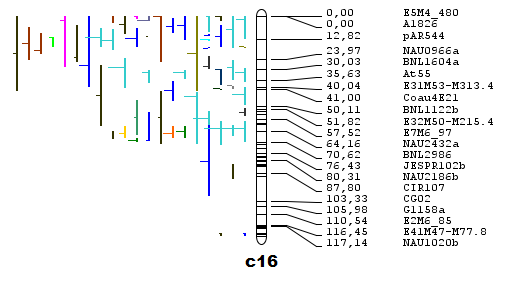

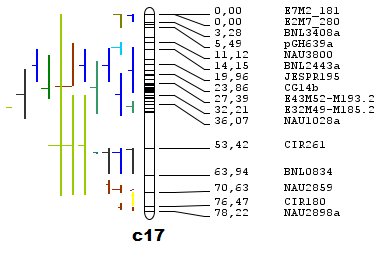

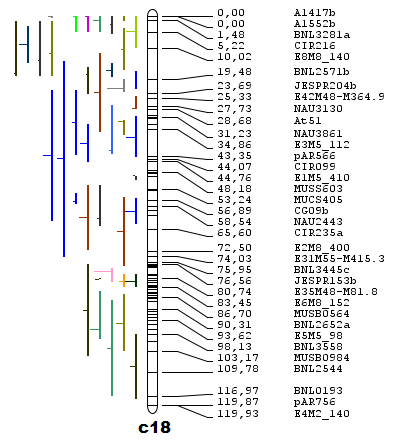

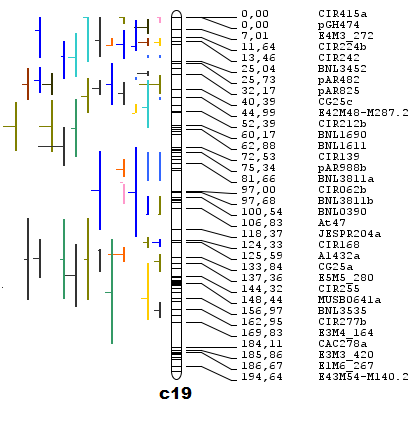

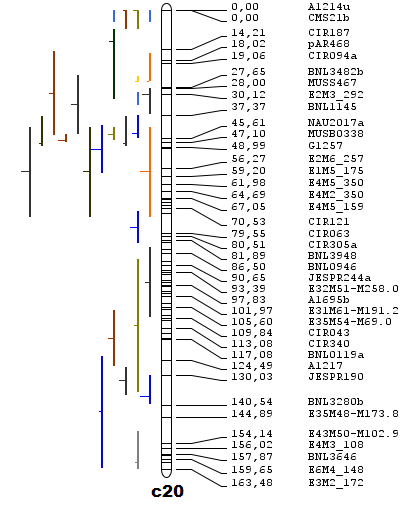

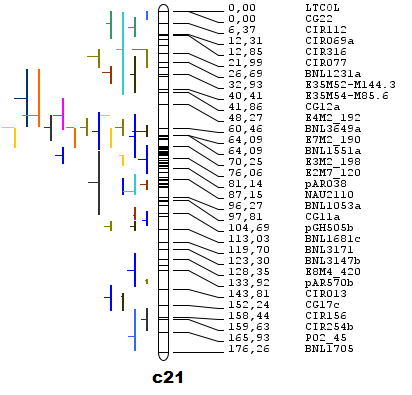

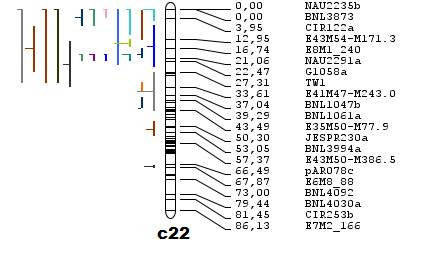

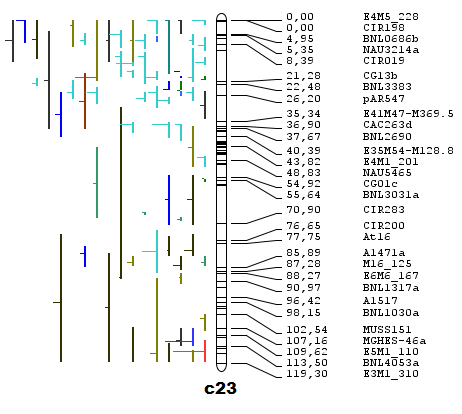

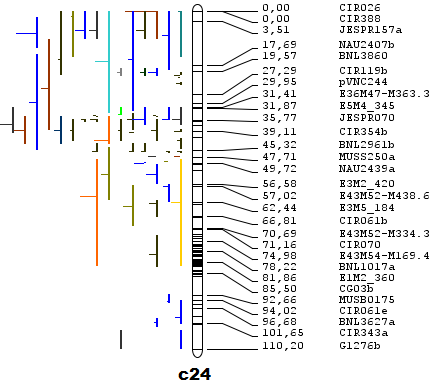

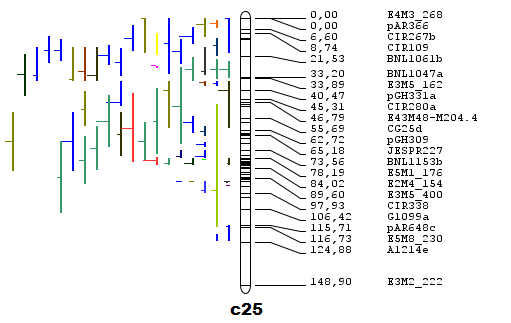

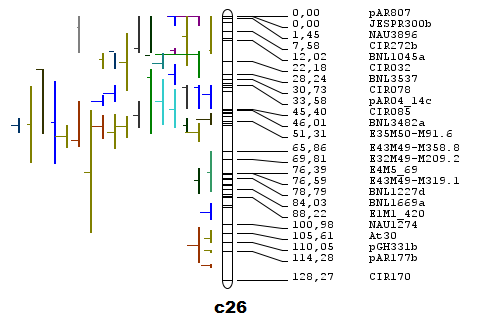
**

**
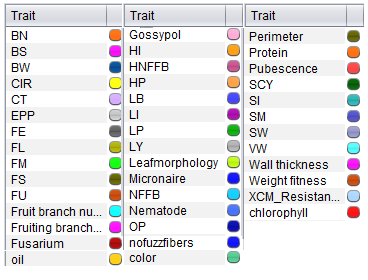
**

**Figure S2: Meta-analysis performed by Biomercator V3**

Figure S2: Below are chromosomes which have been processed using the Biomercator V3 meta-analysis tool. The chromosomes physical sizes were enlarged compared to previous figures to better illustrate locations of clusters represented by shaded regions along the chromosomes. The first cluster on all chromosomes is red, the second, blue, the third green, and the fourth purple. The software and the figures below offer support and confidence to the clusters declared by the manual inference by validating the existence of clusters in the specified regions.


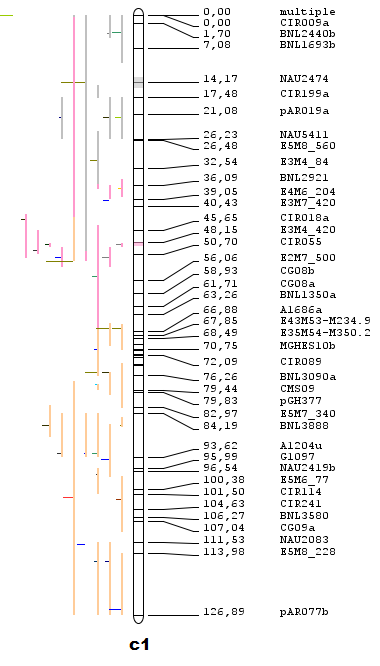

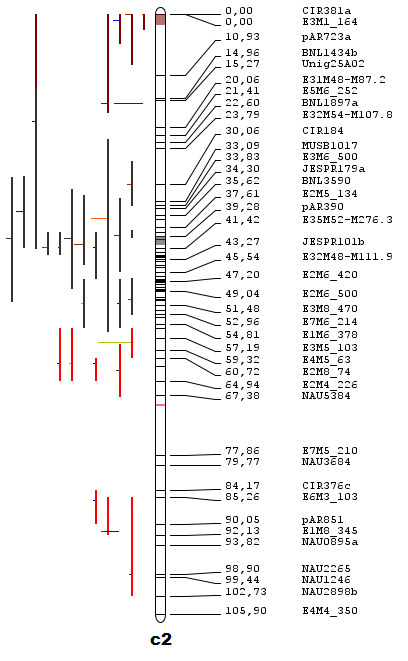

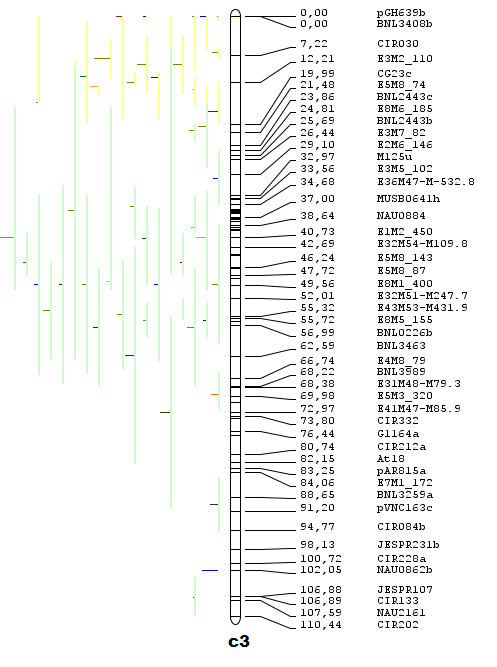

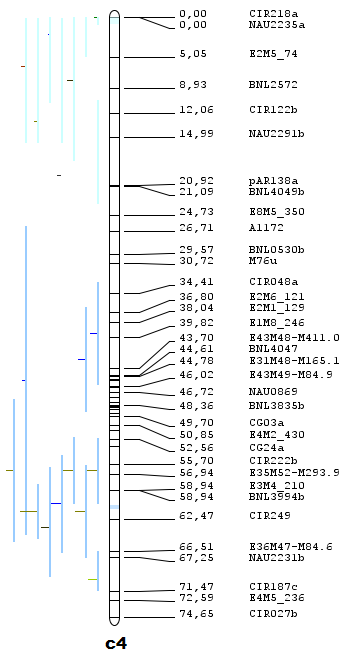

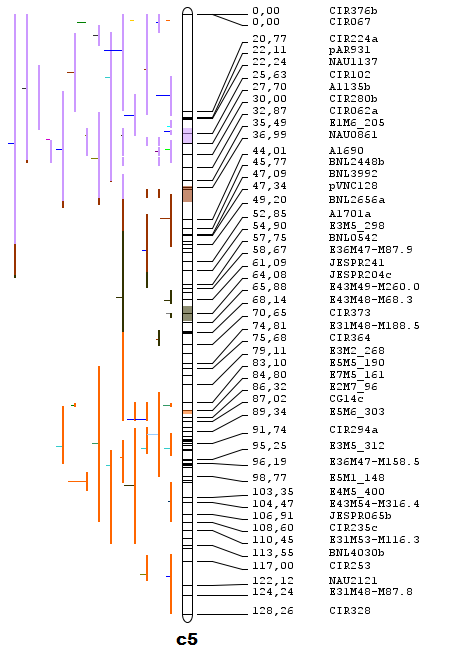

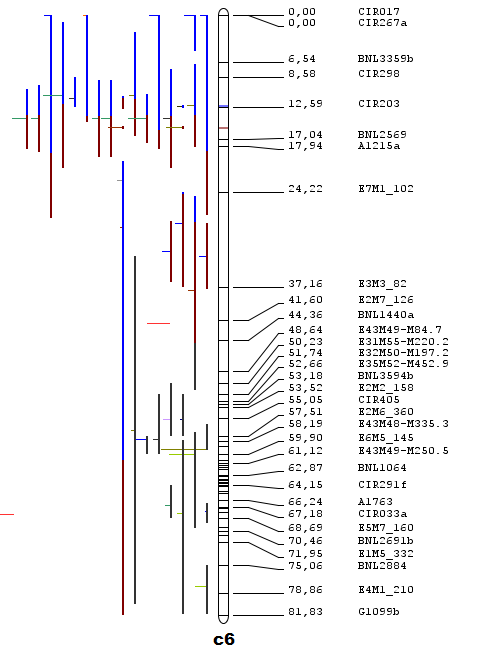

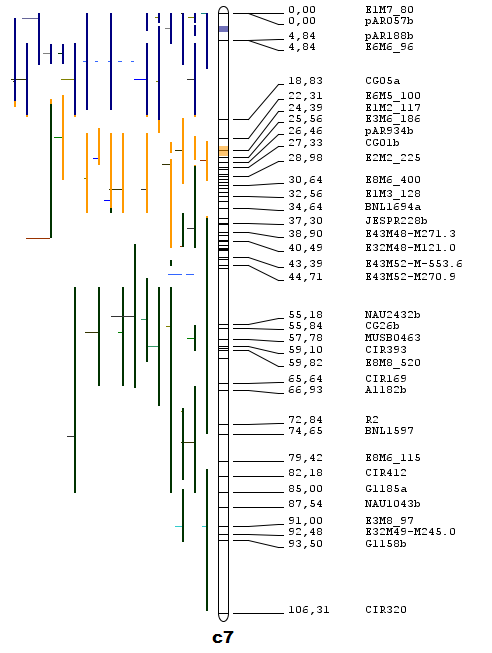

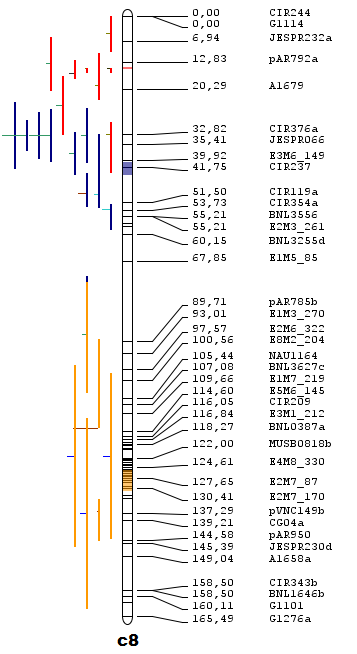

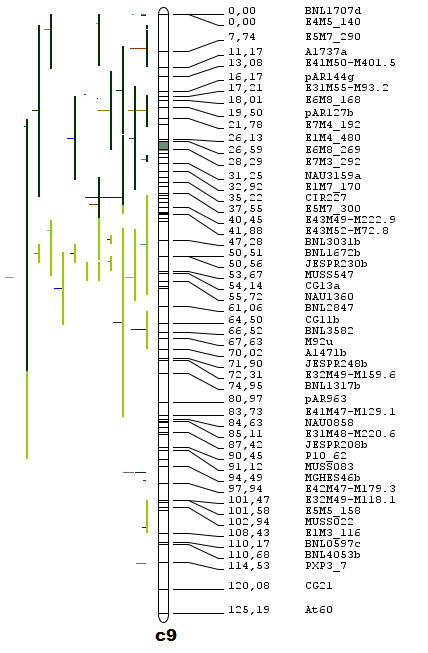

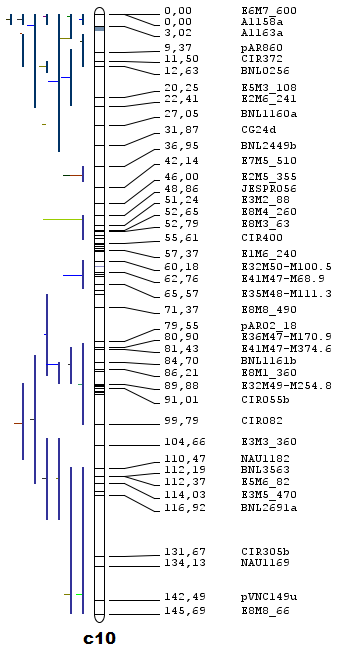

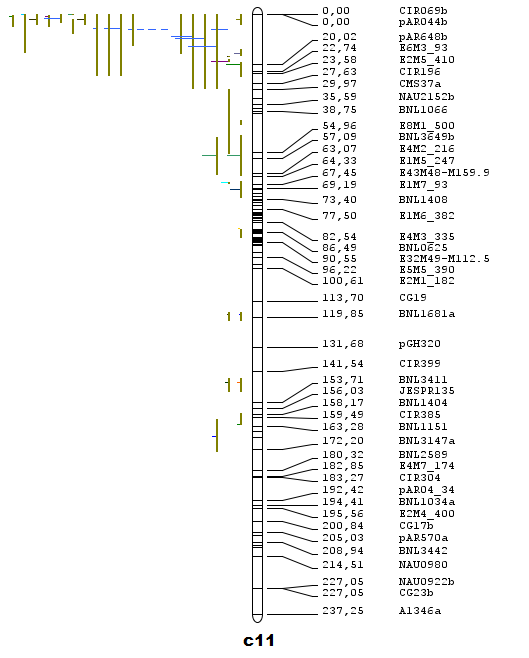

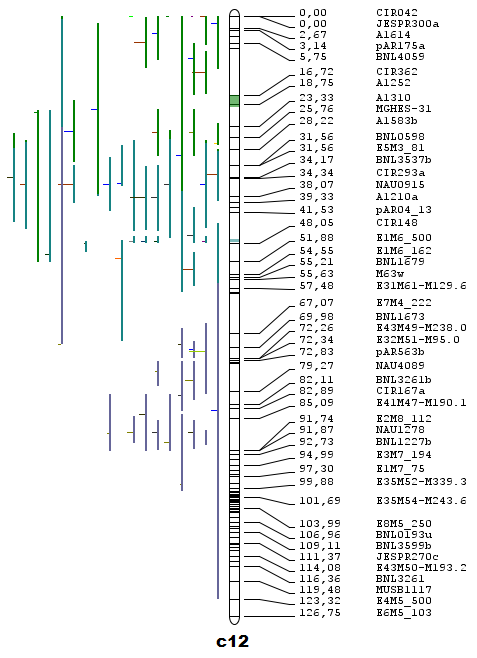

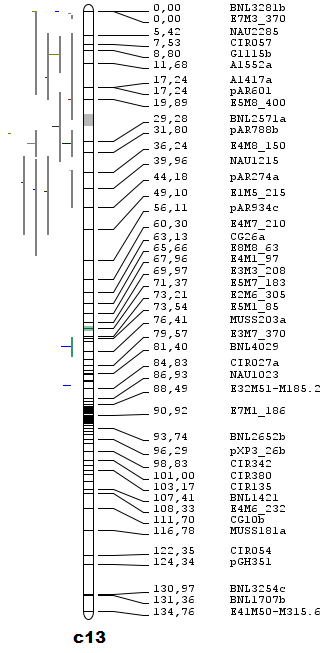

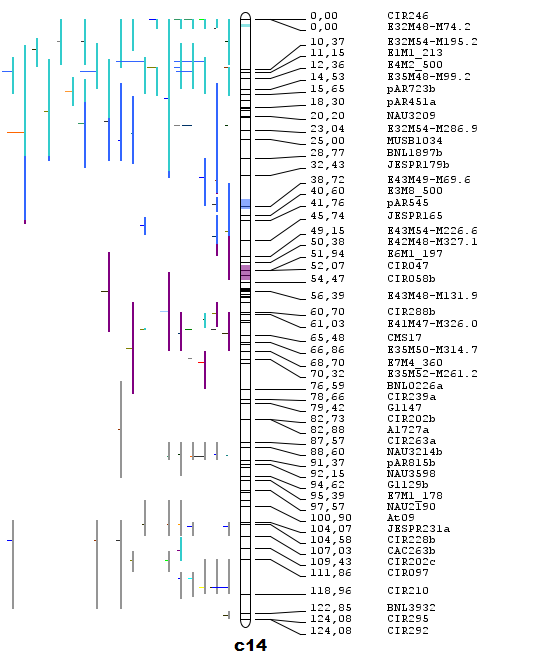

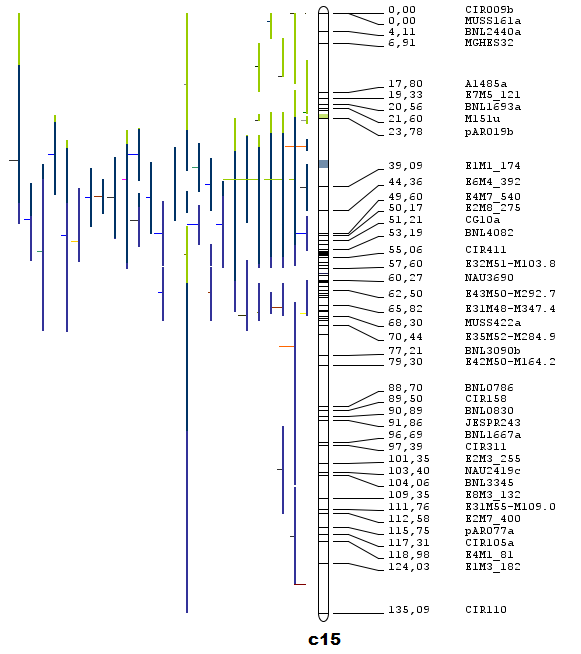

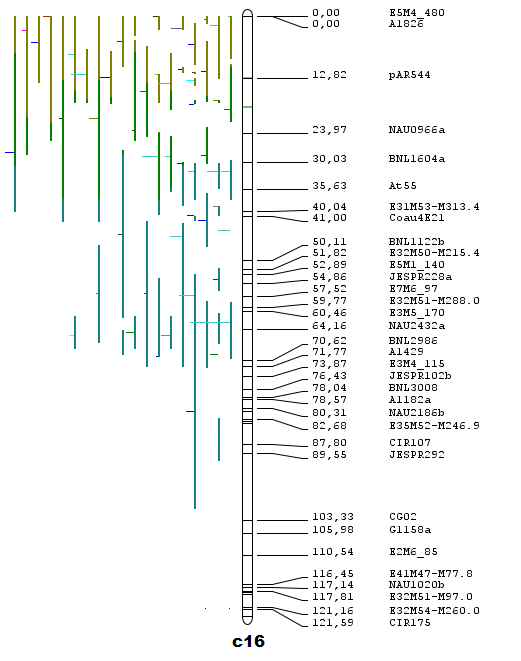

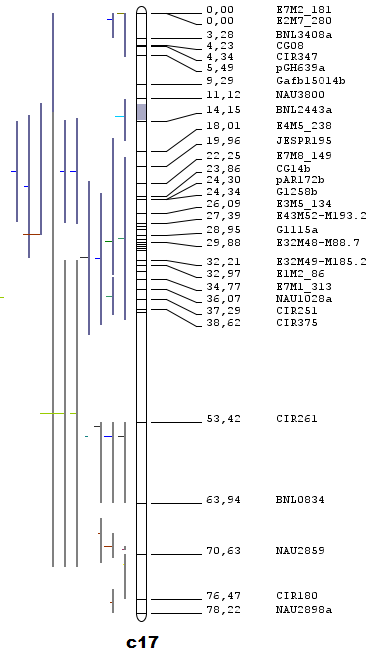

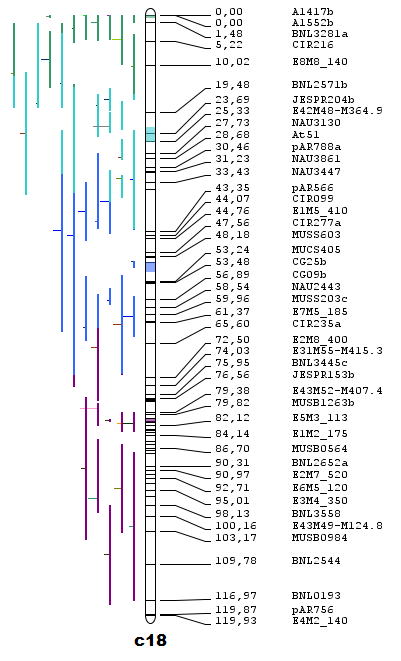

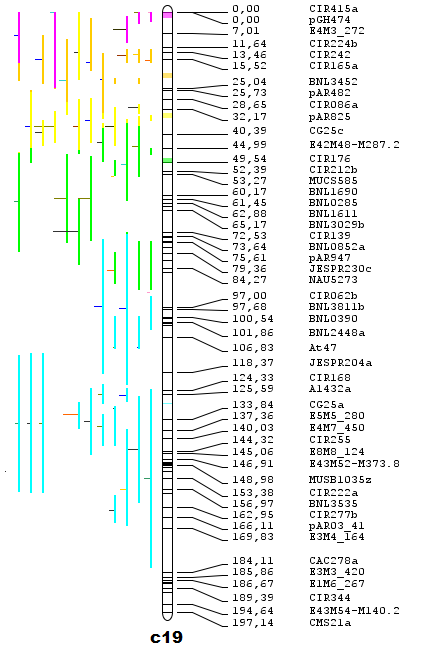

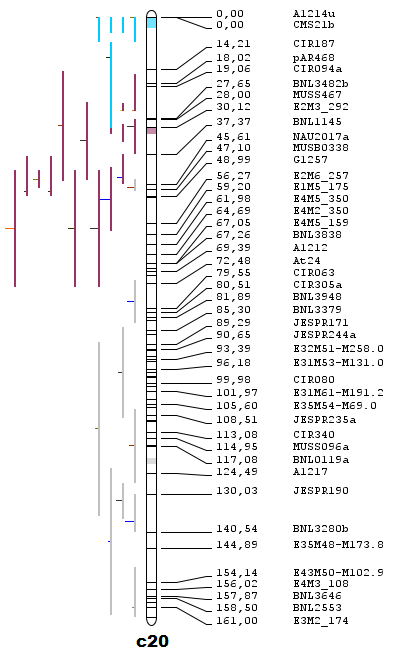

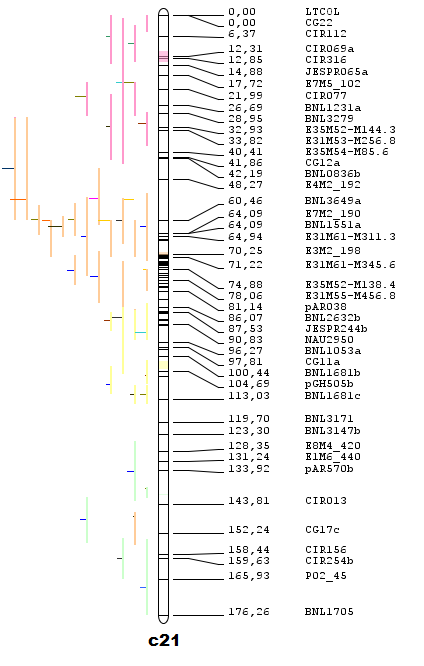

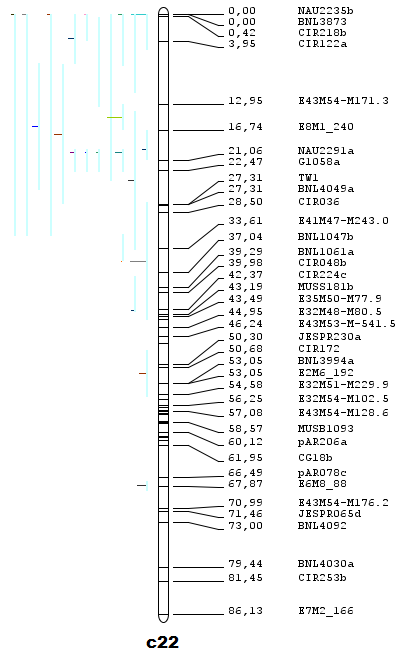

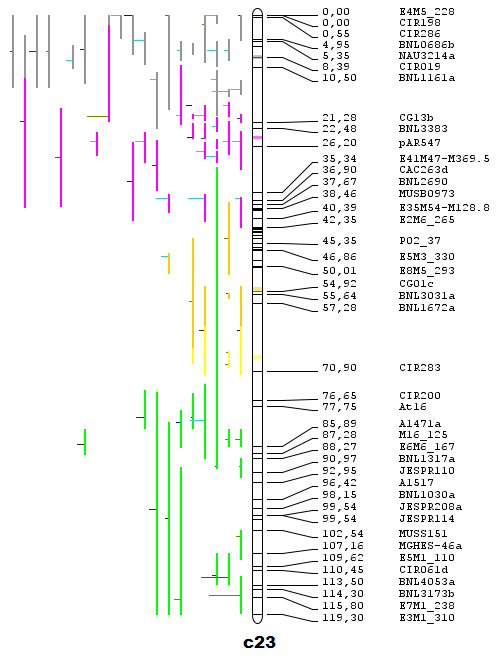

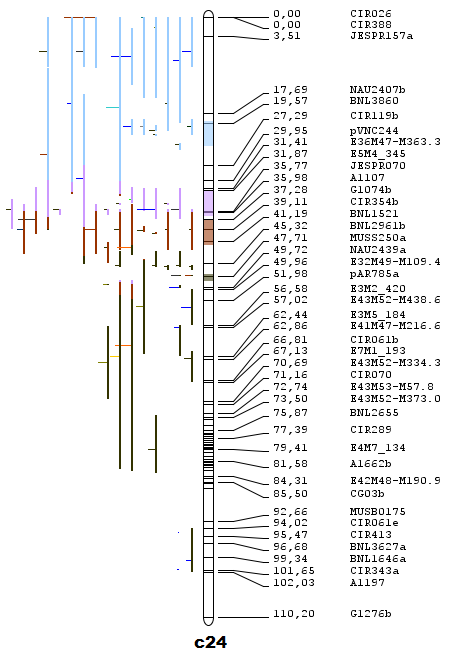

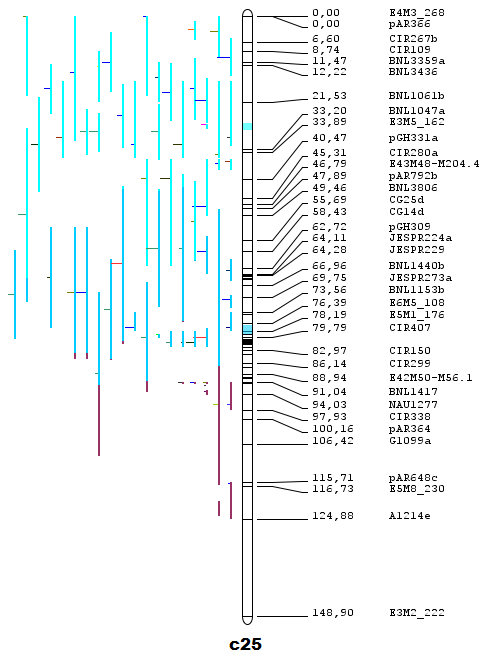

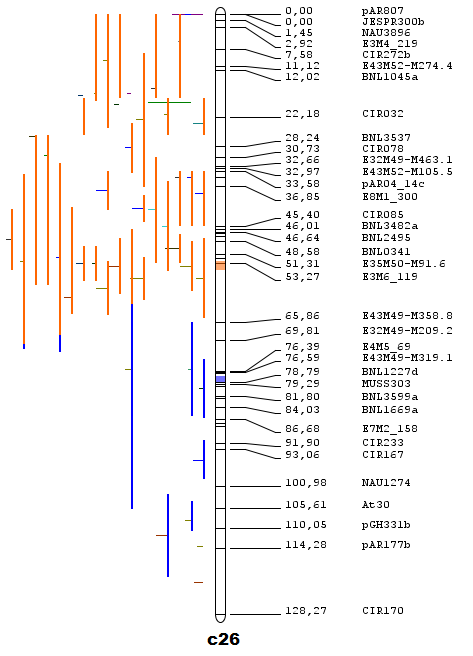


**Figure S3: Fiber strength hotspots present**

Figure S3: Fiber strength QTLs are displayed in purple. Chromosomes with a significant number of QTL which contain hotspots are displayed below.


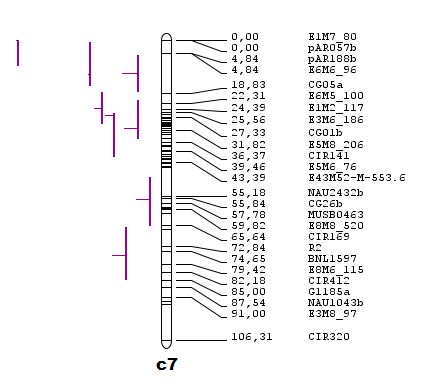

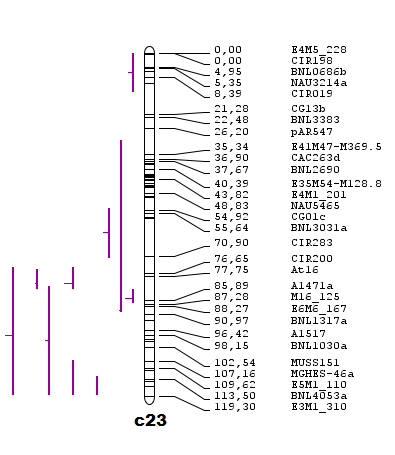

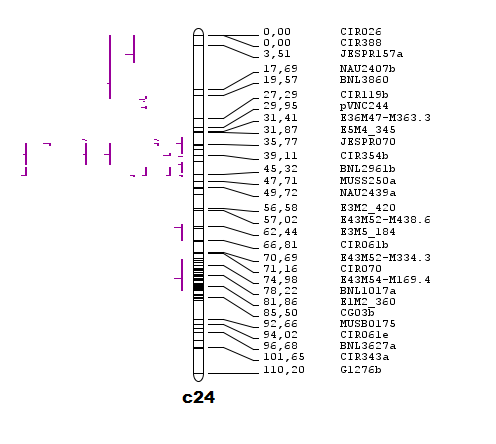


**Figure S4: Fiber length hotspots**

Figure S4 - Fiber length QTLs are displayed in blue. Chromosomes possessing a significant number of fiber lengths QTL that contain fiber length hotspots are displayed below.


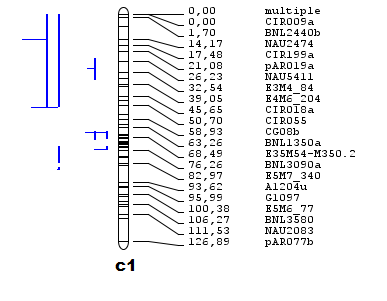

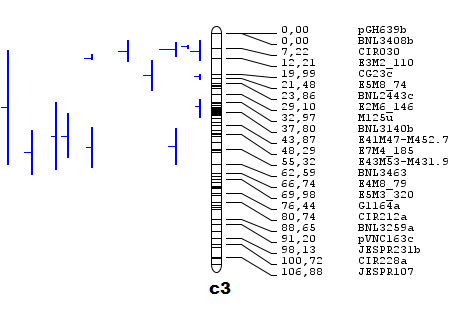

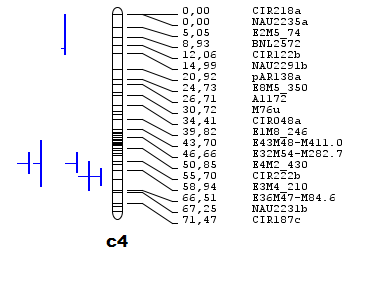

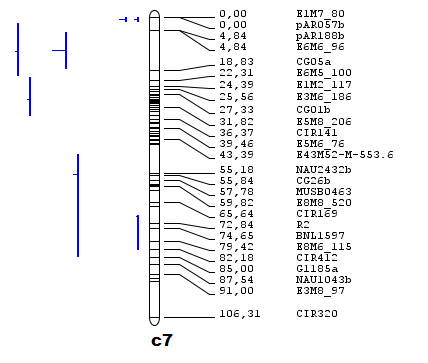

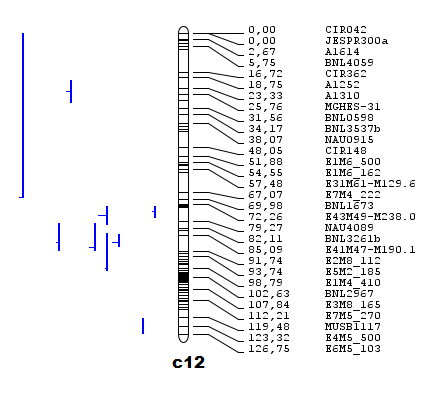

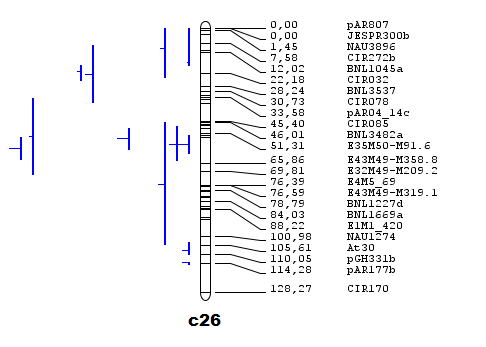


**Figure S5: Fiber uniformity hotspots**

Figure S5: Fiber uniformity QTLs are displayed in orange. Chromosomes possessing a significant number of fiber uniformity QTL contain hotspots and are displayed below.


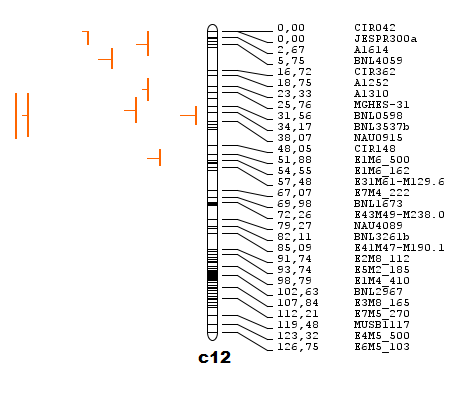

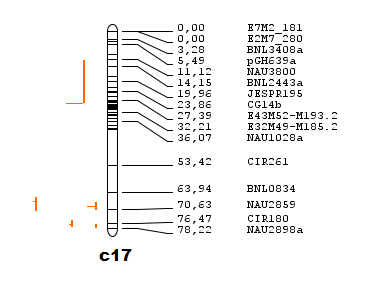


**Figure S6: Fiber elongation hotspots**

Figure S6 - Fiber elongation QTLs are displayed in green. Chromosomes possessing a significant number of QTL may contain hotspots and are displayed below.


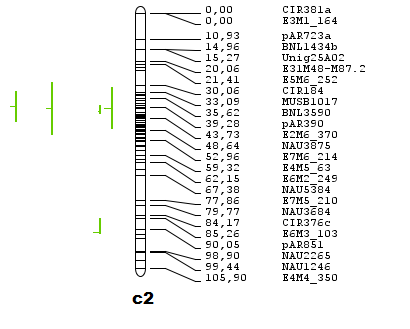

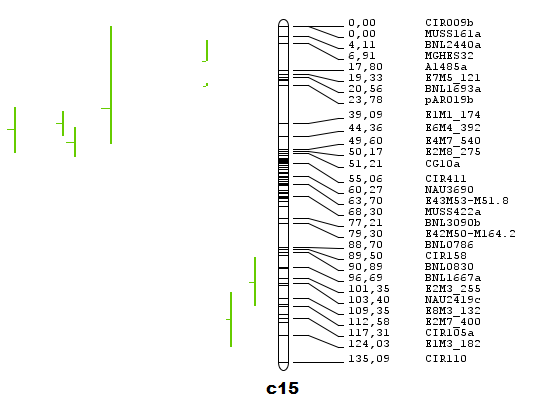

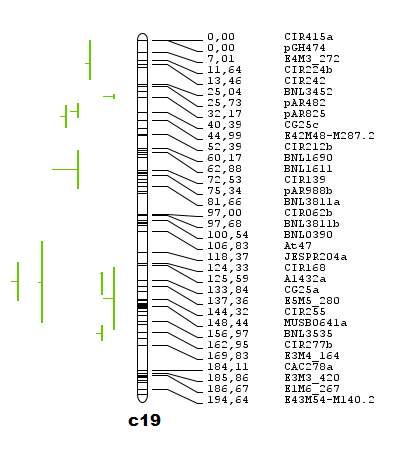

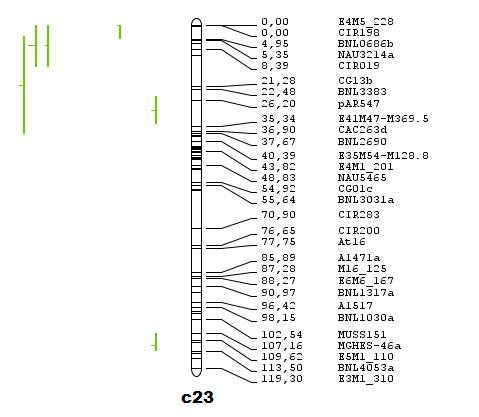


**Figure S7: Micronaire hotspots**

Figure S7: Micronaire QTLs are displayed in red. Chromosomes possessing a significant number of micronaire QTL and contain hotspots are displayed below.


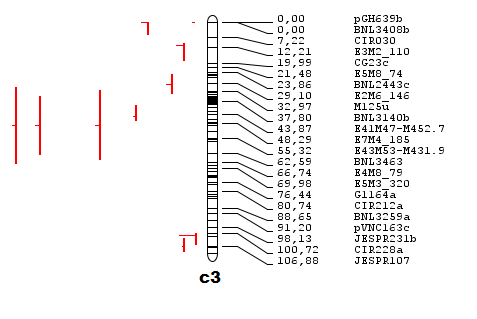

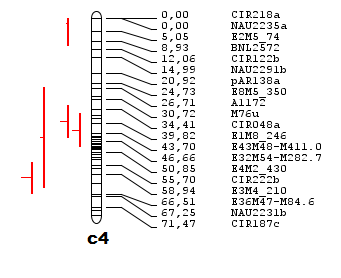

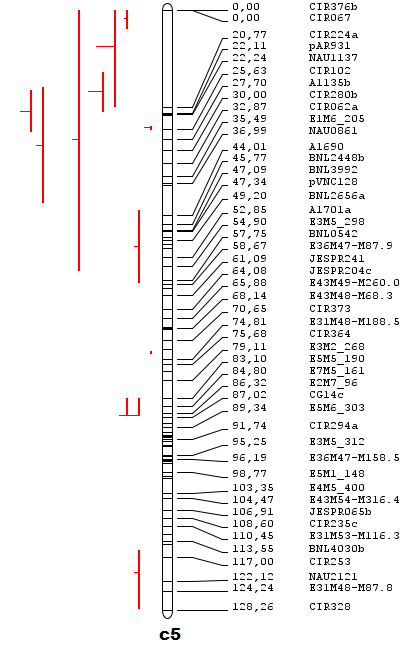

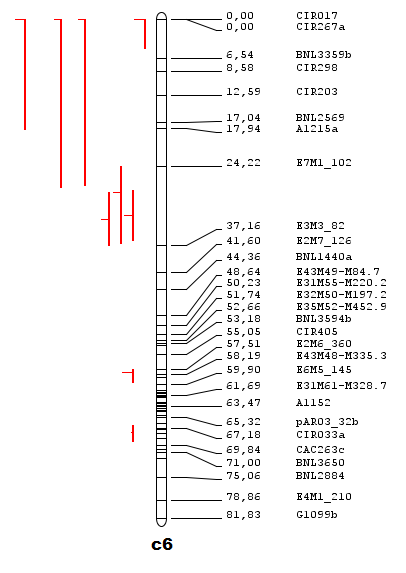

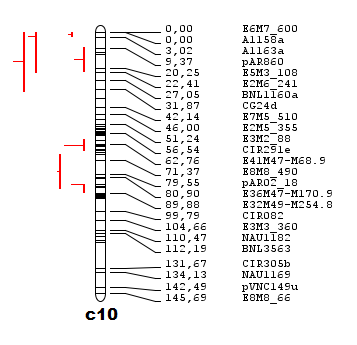

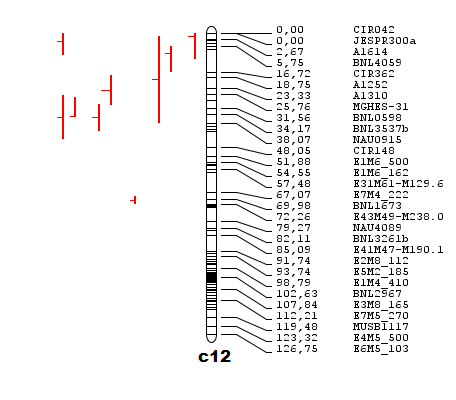

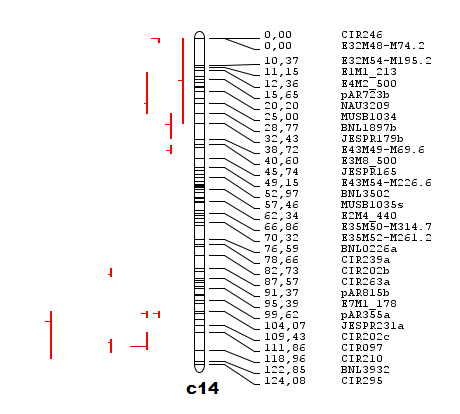

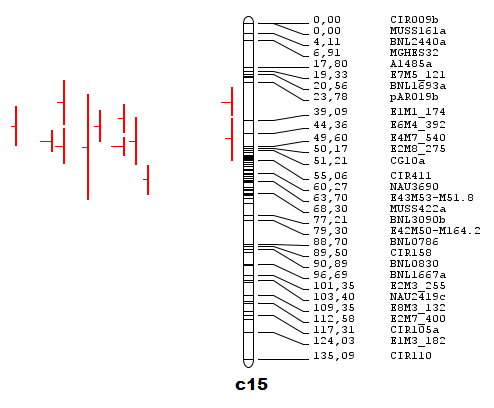

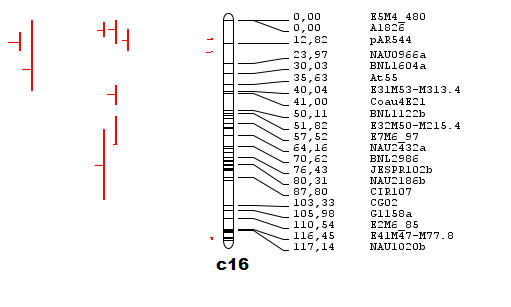

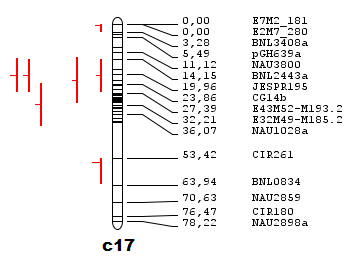

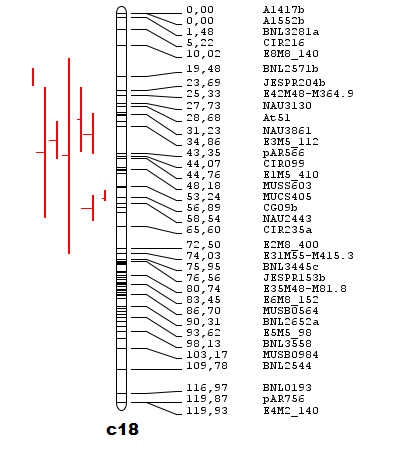

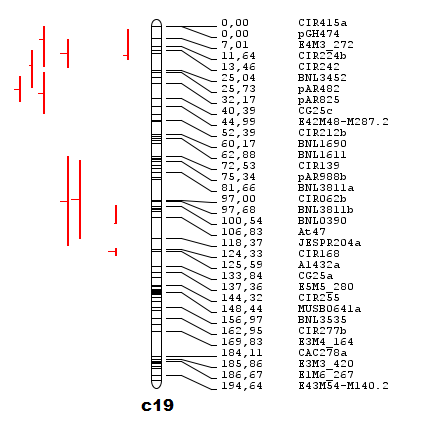

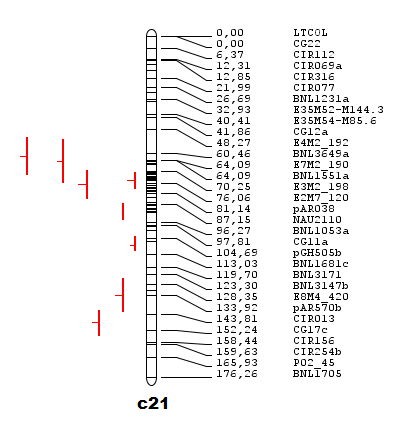

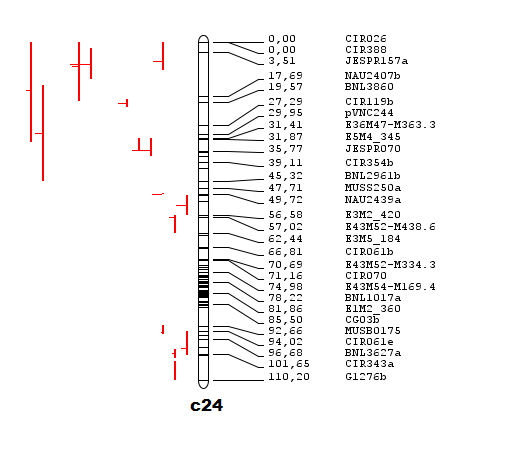

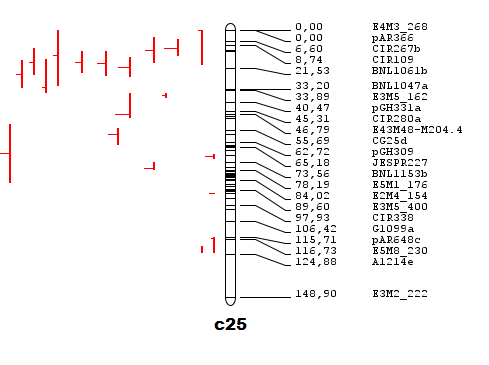

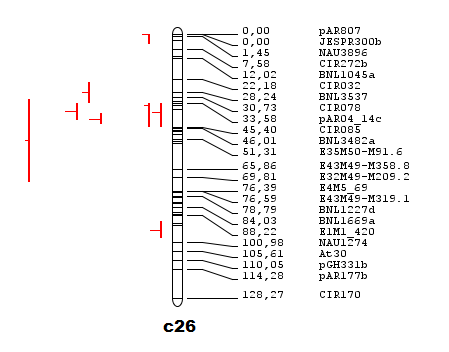


**Figure S8: Color hotspots**

Figure S8: Color QTLs are displayed in pink. Chromosomes possessing a significant number of color QTL and contain hotspots are displayed below.


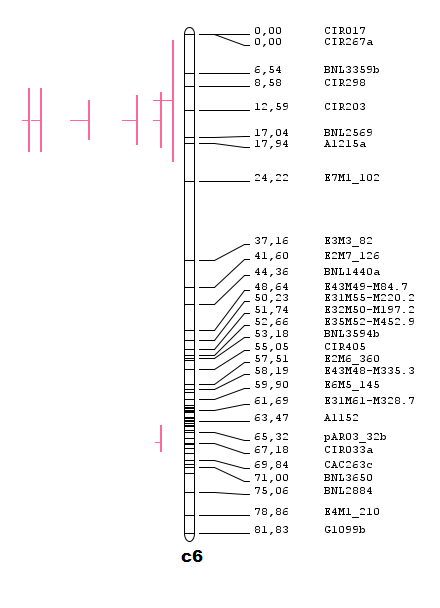

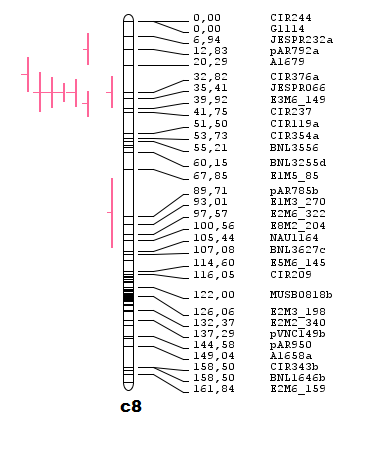

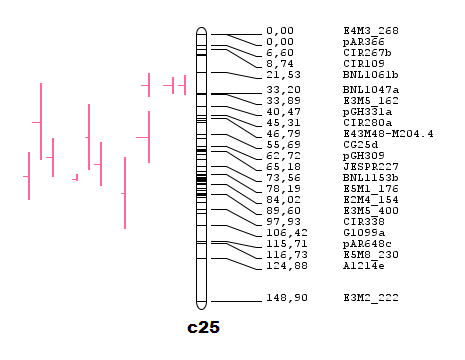


**Figure S9: Leaf morphology hotspots**

Figure S9: Leaf morphology QTLs are displayed in brown. Chromosomes possessing a significant number of leaf morphology QTL and contain hotspots are displayed below.

**
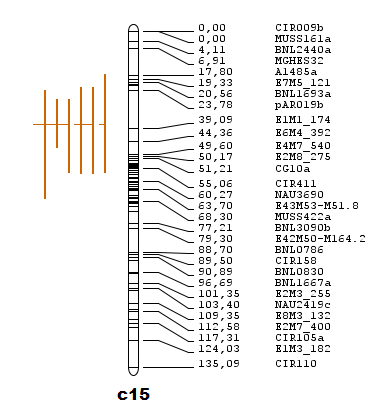
**

**Figure S10: Verticillium wilt resistance (VW) hotspots**

Figure S10: Verticillium wilt resistances (VW) QTLs are displayed in purple. Chromosomes possessing a significant number of VW QTL and contain hotspots are displayed below.


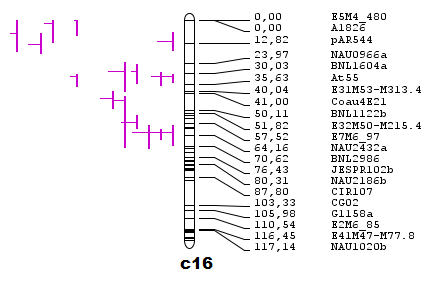

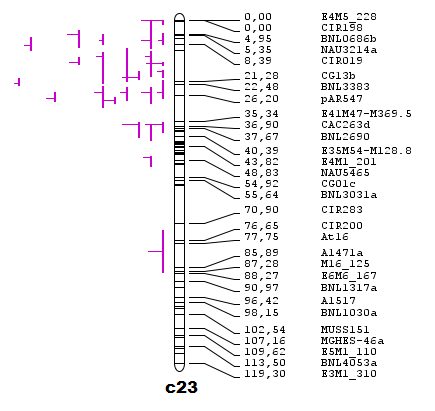


**Figure S11: Nematode Resistance hotspots**

Figure S11: Nematode resistance related QTLs are displayed in dark green. Chromosomes containing a significant number of nematode related QTL and contain hotspots are displayed below.


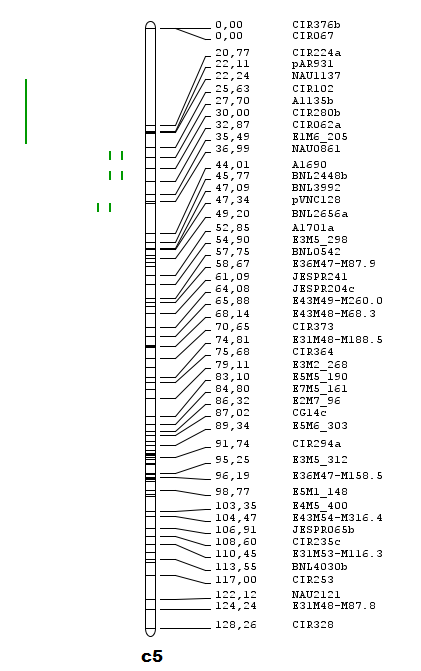

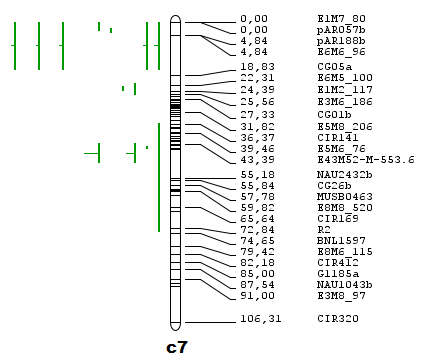

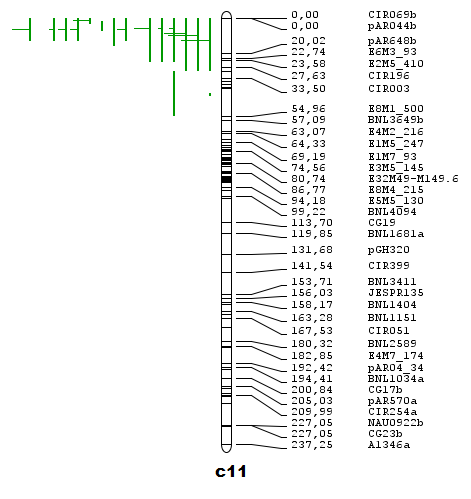

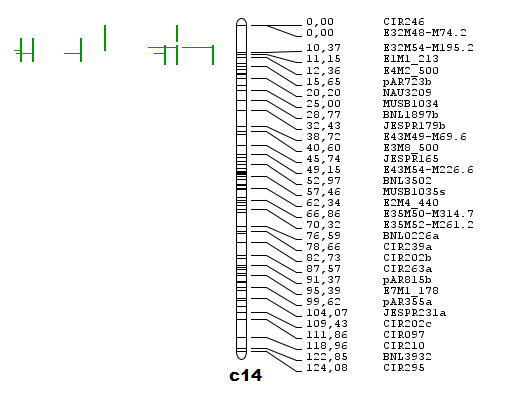

Supplement: Additional file 1: Figure S1 — QTLs and their distribution on the genome. Figure S2. Meta-analysis performed by Biomercator V3. Figure S3. Fiber strength hotspots present. Figure S4. Fiber length hotspots. Figure S5. Fiber uniformity hotspots. Figure S6. Fiber elongation hotspots. Figure S7. Micronaire hotspots. Figure S8. Color hotspots. Figure S9. Leaf morphology hotspots. Figure S10. Verticillium wilt resistance (VW) hotspots. Figure S11. Nematode Resistance hotspots. [file 1471-2164-14-776-S1.docx]
